# Exploring the dynamics of hourglass shaped lattice metastructures

**DOI:** 10.1038/s41598-020-77226-4

**Published:** 2020-12-01

**Authors:** Vivek Gupta, Sondipon Adhikari, Bishakh Bhattacharya

**Affiliations:** 1 Department of Mechanical Engineering, Indian Institute of Technology Kanpur, Kanpur, India; 2 College of Engineering, Swansea University, Swansea, UK

**Keywords:** Engineering, Materials science, Physics

## Abstract

Continuous demand for the improvement of mechanical performance of engineering structures pushes the need for metastructures to fulfil multiple functions. Extensive work on lattice-based metastructure has shown their ability to manipulate wave propagation and producing bandgaps at specific frequency ranges. Enhanced customizability makes them ideal candidates for multifunctional applications. This paper explores a wide range of nonlinear mechanical behavior that can be generated out of the same lattice material by changing the building block into dome shaped structures which improves the functionality of material significantly. We propose a novel hourglass shaped lattice metastructure that takes advantage of the combination of two oppositely oriented coaxial domes, providing an opportunity for higher customizability and the ability to tailor its dynamic response. Six new classes of hourglass shaped lattice metastructures have been developed through combinations of solid shells, regular honeycomb lattices and auxetic lattices. Numerical simulation, analytical modelling, additive layer manufacturing (3D printing) and experimental testing are implemented to justify the evaluation of their mechanics and reveal the underlying physics responsible for their unusual nonlinear behaviour. We further obtained the lattice dependent frequency response and damping offered by the various classes of hourglass metastructures. This study paves the way for incorporating hourglass based oscillators to be used as building block of future mechanical metamaterials, leading to a new class of tunable metamaterial over a wide range of operating frequencies. The proposed class of metastructure will be useful in applications where lightweight and tunable properties with broadband vibration suppression and wave attenuation abilities are necessary.

## Introduction

Metastructures are metamaterial inspired concepts transplanted in structural design. The exploration of metamaterials was ushered in by investigating electromagnetic metamaterials, which exhibited negative permittivity and permeability^1,2^. Motivated by the electromagnetic metamaterials, the concept was extended to acoustic metamaterials^3^, also known as phononic crystals. They are known to exhibit certain types of periodicity or translational symmetry^3^. These metamaterials are engineered to derive their fundamental mechanical properties from the geometry of their structural building blocks, rather than the constituting materials^2^. Unlike the traditional phononic crystals, whose bandgap behavior gets fixed with the patterning of the structural building blocks, the properties of metamaterials are adjustable due to tunability of the local structural resonances of individual elements in the unit cell^4,5^. Since most of the existing metamaterials operate in a narrow band, there is a significant interest in developing tunable metamaterials, which allow for adjustable operating frequencies and have greater potential in acoustic imaging^6^, cloaking^7^ and protection of civil infrastructures from impact and seismic threats^8–10^.

Encouraged by the development of the tunable metamaterials, metastructures have emerged with excellent wave absorption abilities as well as stiffness-to-weight ratio^3,11,12^. It is well proven that the tailoring of geometric and elastic properties of the building blocks of the metastructures could tune its wave behavior^13,14^. Various alterations of periodic arrangements were explored, ranging from a lumped mass model of a 1D metastructure^11^ to cycloidal resonators and an array of cylindrical resonator^15,16^. However, all these metastructure designs have a limited scope of tunability due to the relatively simple geometry of building blocks. A broadband design still requires novel unit cells, which would inevitably increase its tunability. Hence, there is a need to design a unit metastructure with more customizable properties. The lattice based architected metastructures have shown enhanced static properties as well as the ability to control wave propagation, making them ideal for multifunctional applications^17–19^. They opened up new areas of the tunable material property set. This, in addition to evolving 3D printing techniques that enable their manufacturing, have motivated researchers to explore a variety of architectures based on lattice topologies^20–23^,woven topologies^24^, hierarchical structures^25,26^, honeycomb structures^20,27–29^ and foam-like metamaterials^30^.

It can be deduced from the existing literature, that the dome based lattice structures have been rarely explored in metastructure design^31,32^, even though such structures have shown good potential in tuning the stiffness and Poisson’s ratio^33–35^. We have expanded the design space of metastructures by integrating the advantage of lattice geometry with the enhanced tunability of the hourglass shape. Hitherto, this unique combination is not reported in the literature. The shape of the hourglass structure in itself is a fascinating design that contains a combination of two oppositely oriented coaxial domes. For more design complexities, this configuration enables us to consider two standard lattices based on regular honeycomb and auxetic (re-entrant structure). Auxetic materials and structures exhibit an interesting property of negative Poisson’s ratio. By virtue of it, they exhibit synclastic behavior when they are bent, which is deformed into dome shape^31,32^, instead of saddle type deformations, which are typically observed in structures with positive Poisson’s ratio. In addition, auxetic based domes show reduced snap-through in comparison with conventional lattices and solid dome^34^.

The objective of this paper is to develop the foundation of an hourglass shaped latticed metastructure. The basic properties of homogeneous and non-homogeneous classes of unit cell metastructures are addressed. Theoretical and experimental models for understanding the deformation mechanics and mechanical behavior of such structures are presented. Finite element analyses (FEA) of six different hourglass structures have been performed to understand their modal behavior qualitatively. Subsequently, using the mechanics of a local resonator, the vibration attenuation performance has been studied. The tunable stiffness and dispersion curves of a periodically-ranged hourglass oscillator are observed under different topological variations of the unit cell. Finally, a concept of pre-stressing of the hourglass structure has been discussed as a future scope to develop tunable metamaterials with a remotely controlled bandgap using piezoelectric materials.

## Results

**Figure 1:**
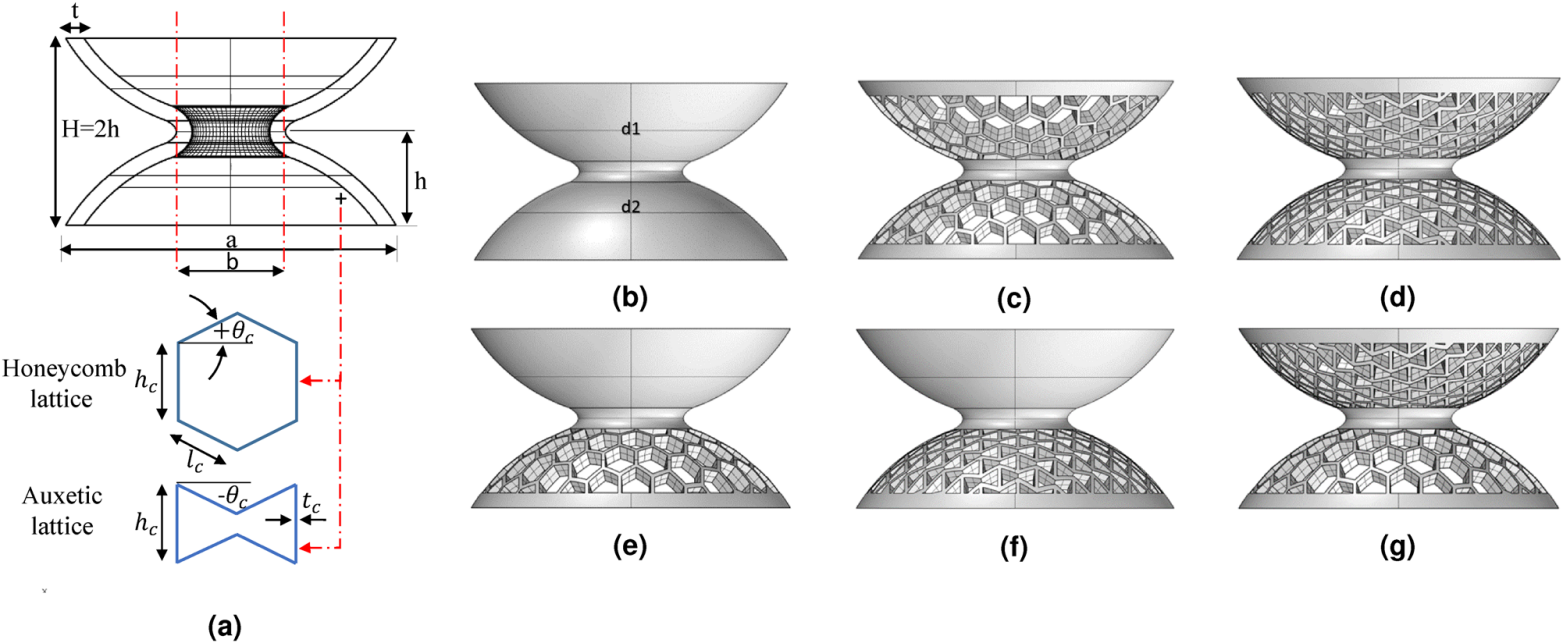
Proposed hourglass shaped lattice metastructures, a combination of two domes d1 and d2 joined by smooth spline surface to avoid stress concentration (**b**–**d**) named as homogeneous (with d1 and d2 domes having lattice symmetry) and (**e**–**g**) non-homogeneous (with d1 and d2 domes having unsymmetrical lattices) categories. (**a**) Schematic of hourglass shape with standard lattices. Homogeneous: (**b**) hourglass as a solid shell (without lattice), (**c**) with regular honeycomb lattices, (**d**) with auxetic lattices. Non-homogeneous: (**e**) combination of regular honeycomb lattice with solid shell, (**f**) combination of auxetic lattices with solid shell, (**g**) combination of regular honeycomb with auxetic lattices.

Figure 1a shows the schematic of the hourglass shaped lattice metastructure. It consists of combinations of two dome shells d1 and d2 (with and without latticed structures) joined by a smooth spline surface to avoid stress concentration. Two cellular topologies are considered here that are standard in practice based on auxetic (re-entrant angle) and regular honeycomb. The structure is modeled mathematically and the nature of deformation is studied experimentally. We have divided the hourglass metastructure into two classes for a comprehensive study: homogeneous (lattice symmetry between d1 and d2 domes) and non-homogeneous (unsymmetrical lattices between d1 and d2) as shown in Fig. 1b–g and the response of different samples have been observed. Moreover, the mechanical behavior of different lattice structures has been studied, corresponding to the double dome formation.

**Figure 2:**
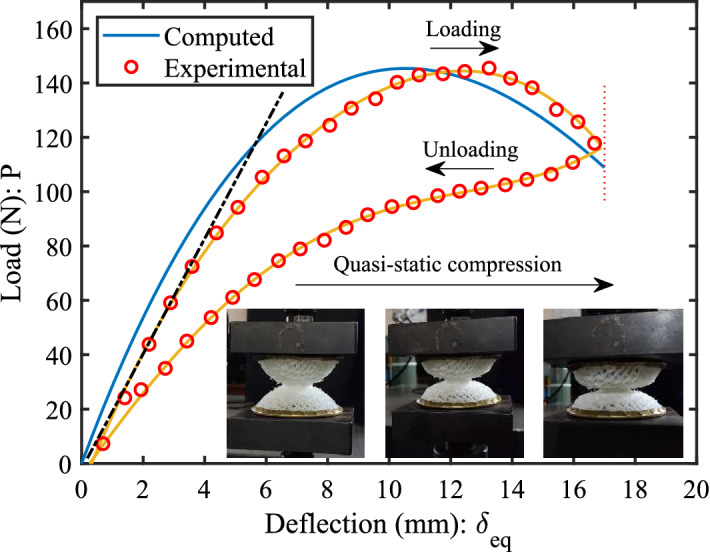
The comparison of static performance is obtained through experiments (tested in UTM with 2kN load-cell) and analytical computations. The hourglass shaped auxetic lattice metastructure (homogeneous) has been considered for attaining load-deflection curves under the loading-unloading test in quasi-static strain rate (0.5 mm/min) condition. The displacement control mechanism is set up to 35% compression from the initial height of the sample.

The auxetic lattices are especially favorable to adapt their shape as perfect domes because they exhibit an interesting property of negative Poisson’s ratio. The nature of deformation allows them to form a dome-shaped double curvature known as synclastic, which is not found in conventional hexagonal lattices^36–38^. An equivalent analytical model of hourglass shaped metastructure has been formulated by considering it as the combination of two coned-disk springs, which can be further treated as the combination of two non-linear spring in series (see supplementary file). The model introduces the fundamental mechanics of load-deflection behavior under the influence of various topological parameters and different lattice geometry. A good agreement between the experimental and analytical results was achieved, as shown in Fig. 2.

The static loading-unloading tests were performed (Instron UTM-1195 with 2kN load cell) under a quasi-static load-deflection relationship. The crosshead speed has been set to 0.5 mm/min (quasi-static strain rate is less than 0.01 s^−1^) to accurately identify the naturally arising transition points. The displacement control mechanism is set at a predetermined strain up to 35% of its initial length of *H* to avoid the effect of plastic zone. The load-deflection curves in Fig. 2 vary linearly up to 3 mm of axial deflection. The line using least-squares fitting with the initial slope of loading gives more than 90% confidence and prediction bound to this limit. Afterward, it becomes non-linear with the 3rd degree of nonlinearity for a particular sample. The hysteresis loss with the retrieval of its original state was observed while unloading the sample. It signifies energy absorption in the loading-unloading process which may vary with the type of lattice. This aspect has already been investigated in detail on the dynamics section through experimental testing, which evaluates the damping performance based on auxetic and honeycomb lattices. The trend obtained analytically follows the same order as obtained through testing.

**Figure 3:**
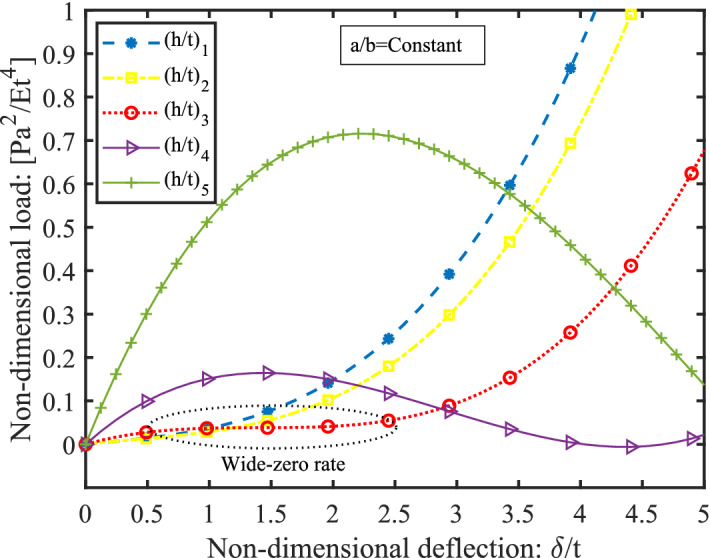
The static nature of the deformation variation (homogeneous hourglass metastructure with auxetic lattice) with different non-dimensional parameters (ratio of the free height of dome *h* and its shell thickness *t*) (*h*/*t*) obtained theoretically. The other geometrical parameters are kept constants such as lattice included angle $\theta _c$ and *a*/*b* ratio. Here, five variations of parameter $(h/t)_5>(h/t)_4> (h/t)_3>(h/t)_2>(h/t)_1$, have been considered and shown with non-dimensional load-deflection axes. The $(h/t)_i$ signifies the ratio of the free height of hourglass *H* to its shell thickness *t* equals to *i*.

This behavior is highly customizable since by introducing suitable variations in the microstructure and prestressing condition, one can observe a phenomenal change in the nature of deformation as has been elucidated in the later section. The load-deflection characteristics of hourglass shaped lattice metastructure vary in an interesting manner with its geometric parameters. The nature of such variation for homogeneous hourglass (based on auxetic lattice) in terms of the non-dimensional parameters is indicated in Fig. 3. Here *P* is the load applied along the axis of the sample, $\delta$ is the deflection along the same direction, *E* is the Young’s modulus of the constitutive material, the other parameters such as *a*/*b* ratio, and lattice angle (re-entrant with ${-\,35}^\circ$) are kept constant. By selecting the values of the parameters judiciously, it is possible to generate the desired nonlinear characteristics corresponding to different values of (*h*/*t*) ratio. One of the critical observations is the sensitivity of the non-dimensional parameter (*h*/*t*) , which may drastically vary the load-deflection behavior. One can produce a wide ‘zero-rate’ range by keeping twice of (*h*/*t*) close to 3. It makes height to thickness ratio of hourglass lattice metastructure equals to three and this is observed the ratio becomes more than 3, the hourglass shaped metastructure can generate negative stiffness, whose magnitude depends upon the lattice topologies (ranging from negative to positive included angle). For a lower (*h*/*t*) ratio, one can achieve an exponential variation of load-deflection characteristics. Such a variation is desirable while developing isolators for variable loading system^39^. An isolator with constant stiffness cannot function satisfactorily in the entire range of varying inertia (see discussion section). In practice, these characteristics are achieved using a combination of steel leaf and rubber spring, which is a complicated design^40^. Similarly, a suitable generation of nonlinear stiffness could widen the suppression band while designing an effective dynamic vibration neutralizer^41^.

**Figure 4:**
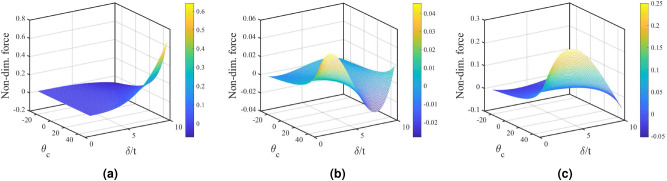
Influence of lattice cell angle: $\theta _c$ with non-dimensional deflection and non-dimensional force obtained analytically for homogeneous hourglass lattice metastructures. The lattice angle varies from negative to positive, which transforms lattice from re-enrant (auxetic) to regular honeycomb, (**a**) represents the variation under (*h*/*t*) ratio equals to unity compared to (**b**) (*h*/*t*) equals to 4, and (**c**) (*h*/*t*) equals to 7.

The influence of lattice cell angle $\theta _c$, on non-dimensional deflection $\delta /t$ is presented corresponding to the variation in the non-dimensional force, as shown in Fig. 4. The lattice cell angle varies from negative to positive, transforming the lattice from auxetic (re-entrant angle) to regular honeycomb. It is observed that the nature of stiffness is non-linear and its variation is significant in the honeycomb lattice region while the same stabilizes for the auxetic lattice region. It also implies that the snap-through buckling is significantly reduced for re-entrant auxetic lattices in comparison to the honeycomb lattices, as shown in Fig. 4b. The existing literature recognizes the instability issue of domes due to snap-through buckling, which can be resolved by introducing the auxetic lattice structure^34^.

**Figure 5:**
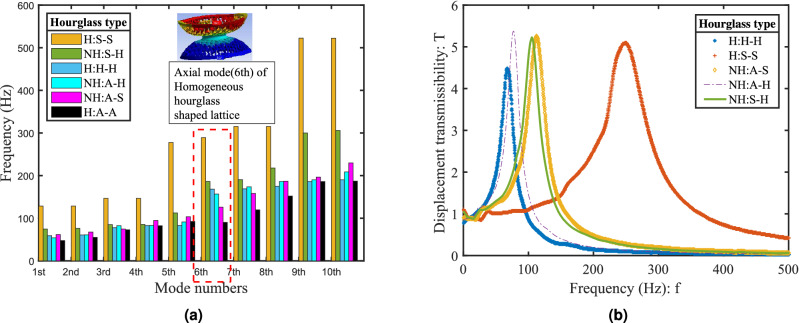
All 3D printed hourglass shaped lattice metastructures were simulated and tested on laser doppler vibrometer (LDV) under base excitation using pseudo-random signal (1600 FFT lines). Homogeneous: (H: S-S solid shell, H: H-H honeycomb, H: A-A auxetic) and non-homogeneous: (NH: S-H solid shell with honeycomb, NH: A-H auxetic with honeycomb, NH: A-S auxetic with solid shell). (**a**) FEA results: Comparative bar chart for first ten modal frequencies obtained from finite element analysis simulations (modal analysis). (**b**) Experimental results: Displacement transmissibility plots (homogeneous and non-homogeneous lattice structure) obtained under frequency response function (FRF) analysis.

**Figure 6:**
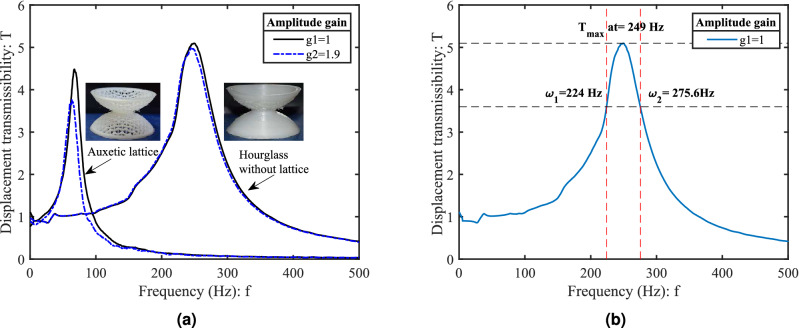
The displacement transmissibility plots (under gain g1(unity), and g2) obtained experimentally (tested in LDV) using the data of frequency response function (FRF). 3D printed hourglass metastructures subjected to base excitation with pseudo-random signal (1600 FFT lines) under the frequency range 0–500 Hz. Homogeneous hourglass metastructure: (**a**) with auxetic lattice and with solid shell (without lattice). (**b**) Half power bandwidth method (− 3 DB points on transfer magnitudes) for calculating the damping factor (shown an example for homogeneous solid shell).

To understand the dynamics of the hourglass and dome-shaped lattices, and their combinations, a detailed experimental study has been conducted to classify the metastructures in terms of vibration response characteristics. We have obtained displacement transmissibility plots for all homogeneous and non-homogeneous samples (Fig. 1b–g) experimentally, as shown in Fig. 5b. The experimental results suggest that the natural frequency of developed hourglass metastructure is sensitive to the changes in microstructural lattice topology. The observed peak on the curves at a particular frequency is representative of the corresponding metastructure’s natural frequency. The lowest natural frequency is observed for homogeneous auxetic metastructure (67.1 Hz), while the highest corresponds to homogeneous solid shell hourglass (250 Hz). Using the “half-power bandwidth method” around each peak, damping for each sample has been calculated (for method see supplementary information) as shown in Fig. 6b. The highest and lowest damping are observed in the case of homogeneous auxetic lattice metastructure with the damping ratio $\zeta =0.125$ and the non-homogeneous solid shell metastructure with honeycomb lattices with $\zeta = 0.0997$ respectively. The damping observed in the other metastructures are non-homogeneous honeycomb and auxetic lattice with $\zeta =0.1057$ followed by the non-homogeneous solid shell with auxetic lattice $\zeta =0.1042$ and homogeneous solid shell (without lattice) with $\zeta =0.1033$ respectively (see Supplementary Table S3). Thus, 21% increment in $\zeta$ value is observed in homogeneous auxetic hourglass metastructure than the homogeneous solid shell metastructure (without lattices). For the same hourglass sample with selected deformation amplitude and frequency range, auxetic lattice structures have higher damping capability than the regular honeycomb lattice. We have obtained a good agreement of transmissibility experiments on laser doppler vibrometer (LDV) with the finite element analysis (FEA) simulations. Modal analysis has been performed for the first ten modes, and corresponding modal frequencies have been extracted through the numerical simulations, as shown in Fig. 5a. The sixth mode (eigenvalue) is found along the axis of hourglass metastructure, which can be directly associated with the natural frequency of each sample. The transfer function responses for each sample are analyzed under different gains g1(unity), g2, g3, g4 using the frequency response (FRF) data obtained through laser doppler vibrometer up to 500 Hz of the frequency range (see supplementary file). The study suggests that the non-linear effect is insignificant for lower strain values, and all metastructures behave as linear under vibration transmissibility experiment. Notably, the homogeneous auxetic and homogeneous solid shell metastructures are affected significantly at higher gain than the other tested samples (Fig. 6a).

**Figure 7:**
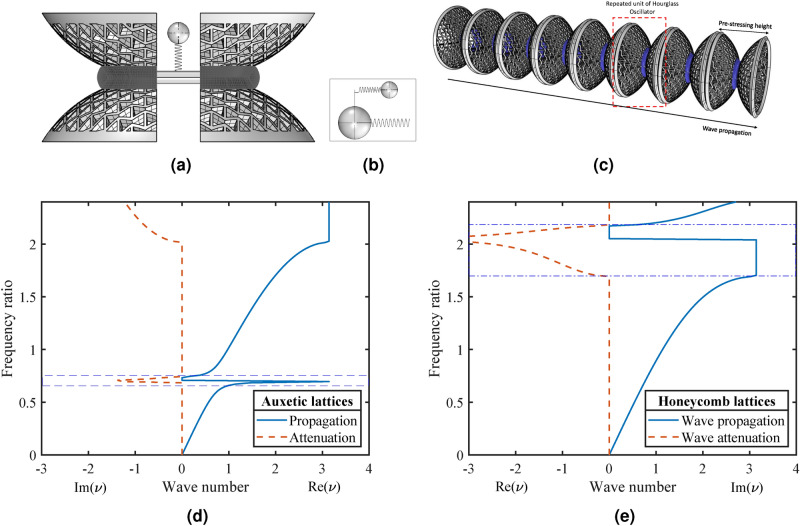
(**a**) The conceptual representation (cross-sectional view with cutting plane is along the axis) of an hourglass oscillator with internal resonating mass (secondary) coupled with an outer metal ring (primary mass). (**b**) Spring-mass system, which is equivalent to hourglass lattice metastructure, spring part is highly customizable stiffness varies from linear to non-linear. (**c**) A basic design of tunable metamaterial with a periodically-ranged hourglass oscillator. Dispersion curves of the tunable metamaterial with different lattice dependent stiffness metastructure (**d**) auxetic based, and (**e**) honeycomb based.

### Introducing the hourglass oscillator

The hourglass lattice metastructure periodically arranged with *n* numbers of oscillators may be treated as locally resonant metamaterials. The dispersion analysis based on 1D diatomic system with a second-order lattice based oscillator was carried out (see supplementary file) mathematically. The conceptual representation of the hourglass based oscillators and the corresponding periodically ranged metamaterial is shown in Fig. 7a,c. In order to tune its bandgap behavior, we have introduced a concept of prestressing of hourglass shaped metastructures to get desired non-linear stiffness behavior. This tuning of the bandgap is also observed using lattice-dependent stiffness obtained experimentally, as shown in Fig. 5b. An equivalent 1D diatomic, locally resonant metamaterial has been considered for initial investigation. The dispersion curves for hourglass based metamaterial with internal resonators having mass ratio $$ m_r=0.125$$, stiffness $$ k_{eq} =$$ 50 N/mm for auxetic and $$ k_{eq}=266$$ N/mm for honeycomb oscillator, has been obtained. It is found under various gains that the transmissibility response does not vary significantly for small deflections (Fig. 6a). Hence, to avoid further complexities, we have considered linear stiffness, obtained experimentally. A solution of the dispersion relationship for harmonic wave motion, i.e., for assigned values of frequency, provides the real and imaginary parts of the wavenumber $$\mu $$ (in Fig. 7d,e). The $$\Omega _r=\omega _r/\omega _0 $$ and $$ \Omega =\omega /\omega _0$$ can be tuned to obtain attenuation over the desired band, without any constraints imposed by wavelength^3^. For low-frequency attenuation, auxetic lattice-based hourglass, and for a higher range of frequency, honeycomb lattices-based hourglass is found to be effective. However, an optimized non-linear stiffness of hourglass metastructure can be obtained by tuning its (*h*/*t*) ratio and $$ \theta _c $$ to widen the bandgap. Such metastructure may work as a building block of damped non-linear phononic materials, which can have the potential applications to enable tunable filters, waveguides, resonators, frequency isolators, and acoustic diodes^3^.

## Discussion

**Figure 8:**
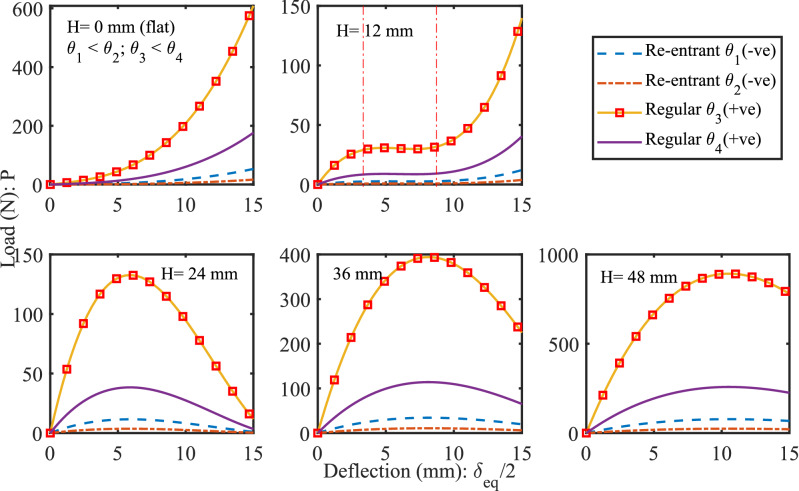
Profiles of load-deflection characteristics obtained theoretically under different undeformed heights (*H* equals to combined heights of d1 and d2 lattice domes) of hourglass shaped lattice metastructure. The variation of lattice angle from negative to positive transforms lattice from auxetic to honeycomb, influencing load-bearing capacity. At $$ H=0$$ mm represents an initially flat metastructure, at $$ H= 12$$ mm generates a curve with **“wide zero-rate range”** where $$ k_{eq} $$ becomes zero for a wider range of deflection, at $$ H= 24$$ mm metastructure generates positive and negative $$ k_{eq} $$, at $$ H= 36$$ mm as d1 and d2 dome radii increases along with dome angle $$\phi $$ generate a wider third-degree profile with $$ k_{eq} $$ becomes zero for shorter range, at $$ H= 48$$ mm generates a wider profile with higher load-bearing capacity.

Notable manifestation of engineering science was involved in the construction of roman domes/arch architectures^42,43^. The central idea that makes the awe-inspiring structures possible is based on simple Newtonian physics. The interplay of gravitational force and the equilibrium forces cause compression on particular segments of the dome. Moreover, an arch/dome lets us redirect forces in a way that pushes towards its center of gravity for discreet blocks or lattices. In this study, the lattices are introduced in a dome shaped curvature to enhance their functionality due to compression forces. The hourglass-shaped lattice metastructure that takes advantage of the combination of two oppositely oriented coaxial domes, providing an opportunity for higher customizability and the ability to tailor its dynamic response. Enhanced functionality of lattices has been achieved through the careful design of the metastructure. Different non-linear stiffness profiles have been observed in Fig. 8, which are sensitive to its non-dimensional *h*/*t* ratio and lattice cell angle $\theta _c$. The influence of lattice thickness can also be perceived by considering (*h*/*t*) parameter where shell thickness *t* may be viewed as lattice thickness. The analytical computations provided in the supplementary material explain *h* as free height (undeformed height) of truncated sphere formed by the upper or lower surface.

Here, $h=0$ implies that the free height of the spherical surface is null and it became flat circular sheet of thickness *t*. Hence, the overall height of hourglass metastructure *H* (taken twice of *h*, Fig. S2 and supplementary method section) becomes zero. This may be theoretically considered as a condition where both the spherical shells of the hourglass structure transform into flat circular surface with thickness *t*. The lattice dependent natural frequencies have been observed in Fig. 5b experimentally. The load-deflection responses for a small value of the undeformed height of hourglass, *H* is non-linear and exponential (Fig. 8), which is important for applications where the natural frequency of the system is required to be constant over a wide range of applied load^40,41^. The undeformed height *H* of all samples is 48 mm. From the amplitude gain results, it is evident that the nonlinear effect is insignificant at lower strain values. It may be substantiated from the analytical results obtained for different undeformed heights *H*, as shown in Fig. 8, moreover, for height, nearly 12 mm produces a wider-zero rate range with zero stiffness showing significant nonlinear effects at lower strain values. This range of height is suitable for designing non-linear oscillators. Coupling with piezoelectric bimorph offers an exciting aspect of switching the stiffness zones (Fig. 8) by pre-stressing the hourglass metastructure. If *n* number of hourglass oscillators are treated as nonlinear phononic material with resonating lumped mass using tunable stiffness, it can produce a wider attenuation zone^3^. The presence of nonlinear terms in the equation of motion does not allow Bloch’s theorem to be invoked in the usual manner, this may be overcome by perturbation approach^44^. The magnitude of load-bearing capacity can be tuned by altering the lattice cell angle from (−ve) re-entrant to honeycomb. It is observed that the natural frequency and stiffness of auxetic lattice based hourglass are less than the honeycomb lattice structure. By providing suitable variations on the re-entrant angle, one can obtain attenuation over the desired band, without any constraint imposed by the wavelength (in Fig. 7). In actual practice, low-frequency attenuation requires low stiffness, which always poses a challenge to the researchers^3^.

In summary, six lattice-based hourglass metastructures have been proposed, analytically modelled and analyzed through experimental and numerical studies to investigate their mechanical and structural properties. The realization of their static and dynamic responses are obtained through the quasi-static compression and transmissibility tests. A lattice-based comparative study has been conducted by classifying the homogeneous and non-homogeneous category of 3D printed hourglass samples. The governing load-deflection relationship has been obtained analytically, which reports the non-linear stiffness variation of hourglass metastructure and its sensitivity to lattice topologies with non-dimensional parameters. The transmissibility tests of all six samples facilitate the lattice-based comparison to obtain the different stiffness profiles and lattice dependent natural frequencies. The auxetic based hourglass samples and their combinations are observed to be less stiff than the honeycomb lattice-based, which shifts its natural frequency to the higher side. The damping values for each sample have been evaluated experimentally. Homogeneous class of auxetic lattice based hourglass structure offers the highest damping compare to honeycomb lattice based samples. The superior functionality of auxetic lattices has been observed by incorporating them into the dome shape of the hourglass structure, making re-entrant lattice suitable for better energy absorption than the other combinations. The first ten modal frequencies and mode shapes were extracted through the numerical simulations. The sixth mode shape is observed along the longitudinal axis of the hourglass metastructure. A close agreement is obtained through the experimental validations.

The emergence of lattice-based different non-linear stiffness profiles and damping values have been estimated through experimental, analytical and numerical simulations for hourglass shape metastructures. Further experimental studies investigating the different lattice functionality with a variety of dome shapes may be required to understand the critical geometric shape responsible for a specific macro-mechanical behavior. Combining these experiments with analytical and numerical studies will allow a structural picture of a customizable mechanical response to be modelled. From this foundation, one can envisage being able to design an hourglass based lattice metastructure as a building block of tunable metamaterial and a novel unit cell for a vibration isolation bed.

We propose the first step towards contemplating this hourglass shape in the lattice-based metastructure design. Looking forward, the hourglass shape offers an exciting prospect for coupling piezoelectric/magnetostrictive materials to tune its mechanical response using controllable electric signal. One can readily envisage the electrical-switching of band-gap behaviour through the inclusion of such smart periodic unit cells.

## Methods

### Materials

**Figure 9:**
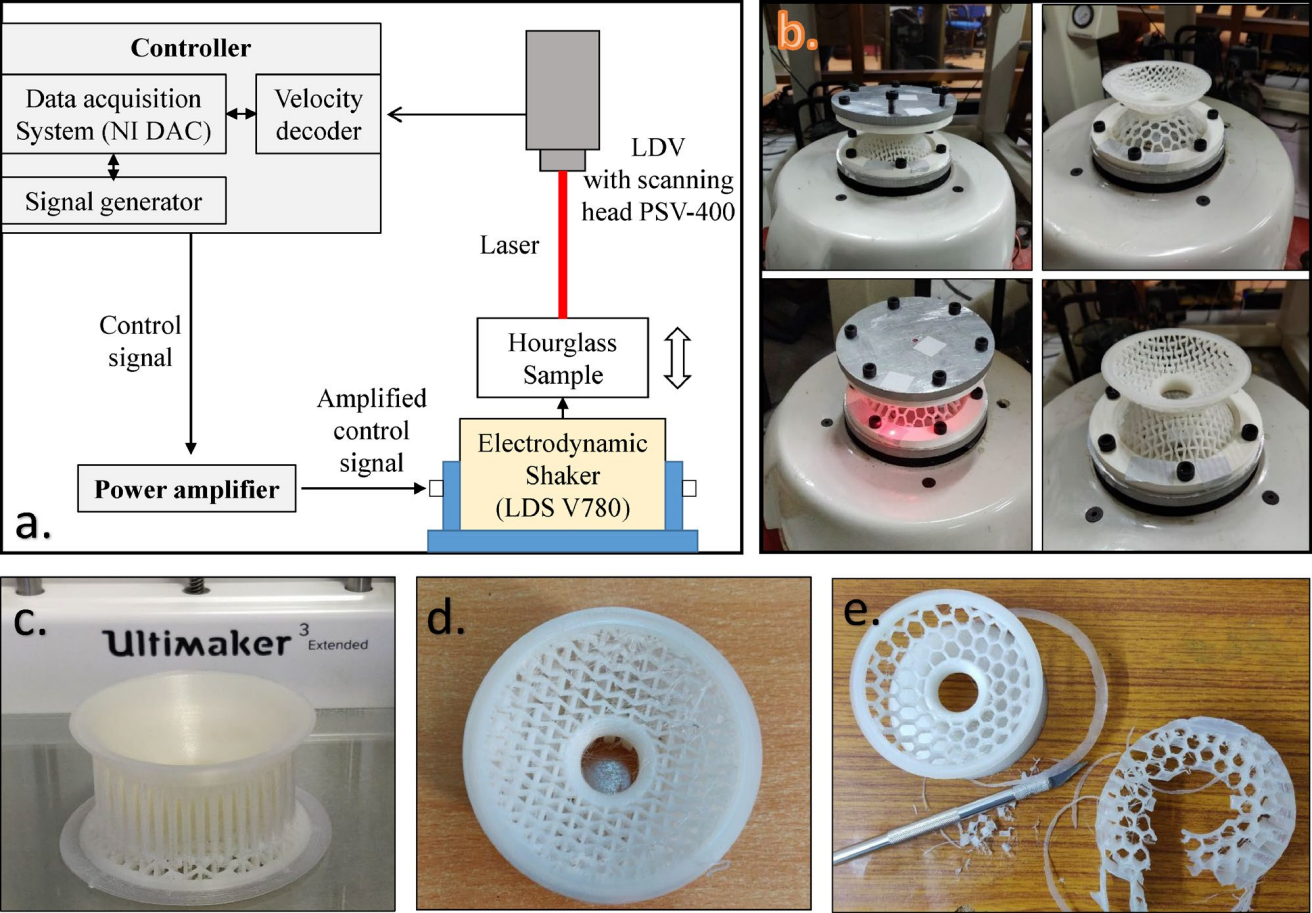
(**a**) Schematic diagram of the experimental setup showing how the vibration transmissibility tests are performed on laser doppler vibrometer (LDV). (**b**) Shows the attachments of 3D printed hourglass lattice metastructures fixed adequately with the base plate attached with electrodynamic LDS shaker and deadweight tightened with the help of fixtures. (**c**) 3D printed sample with support material. (**d**) Top view of homogeneous auxetic hourglass sample after removing all the supports. (**e**) Removing all the supports and cleaning samples with a precision carving cutter.

 A flexible 3D printing material PCTPE (plasticized copolyamide thermoplastic elastomer) provided by Taulman 3D (see supplementary Table S2) is used to print hourglass metastructure as an experimental sample, and high strength PLA (polylactic acid thermoplastic) is used for its fixture. The basic properties of PCTPE material are characterized by the Young’s modulus of $$ E= 75$$ Mpa, Poisson’s ratio $$\nu =0.28$$, and density $$ \rho =0.96$$ gm/cm^3^. An aluminum metal plate with density $$ \rho =2.7$$ gm/cm^3^, radius 50 mm and thickness 8 mm were used as dead weight and base plate for each sample testing setup as shown in Fig. 9.

### Fabrication using additive layer manufacturing

 The modeling of homogeneous (d1 and d2 domes with lattice symmetry) and non-homogeneous (d1 and d2 domes with unsymmetrical lattices) hourglass samples have been fabricated using.

Ultimaker 3.0 Extended multi-material 3D printer. The developed metastructure sample is printed as a whole body with d1 and d2 domes joined by a smooth spline surface to avoid stress concentration (Fig. 1). Each dome is constituted with standard lattices i.e., auxetic, regular honeycomb, and solid shell type. The sphere radius of d1 and d2 domes are 50 mm, dome height (*h*) is 24 mm (makes the undeformed height of the hourglass $$ H= 48$$ mm), radial thickness 4 mm with base radius is 42 mm. Each lattice beam has a thickness of 1 mm (see supplementary file for more details). The 3D printing specification with slicing 0.15 mm layer thickness resolution, 100$$\% $$ infill density with triangular infill pattern, and necessary rectilinear and zig-zag supports (minimum $${45}^\circ $$ overhang angle) were used to prevent the dome from collapsing (see supplementary Table S1). Within the limitation of 3D printing technology, the layer orientation is known to influence the mechanical properties of the material. Therefore, all the samples are printed along the same orientation on the build platform. A standard dog bone tensile testing specimen of PCTPE (with dimensions ASTM 638-14 Type IV recommended for polymeric/plastic samples) with 100% infill density has been developed and performed testing to evaluate the modulus and its ultimate tensile strength (see supplementary Figure S8 and Table S4).

### Static testing

 The measurement of load-deflection characteristics of hourglass shaped (re-entrant auxetic) 3D printed unit cell has been carried out in universal testing machine (Instron UTM-1195 with 2kN load cell). The cross-head speed of 0.5 mm/min (quasi-static) has been set to get exact naturally arising transition points. Furthermore, the data points have been processed and plotted to identify the non-linear stiffness behavior of the sample. The experimental results have been analyzed and approximated with a third and fourth-degree polynomial to estimate the non-linear stiffness from load-deflection characteristics.

### Dynamic testing

 The experimental dynamic responses of hourglass shaped metastructure are obtained using non-contact vibration measurement techniques. A comparative study of all six samples has been performed using 3D laser doppler vibrometer (LDV) from Polytec, mounted on a tripod, is used to measure the velocity along the beam, and the NI DAC systems are used for data acquisition and signal processing. A retro-reflective tape is applied to the top and bottom plate (under which hourglass samples are sandwiched), enhancing its ability to reflect the incident laser beam. See Fig. 9 for the experimental schematic (see supplementary for detailed information). The displacement transmissibility tests are carried out using base excitation under the pseudo-random signal (1600 FFT lines) and sine sweep, performed by LDS-electrodynamic shaker (V780). The frequency response function data (FRF) up to 500 Hz were analyzed under different gains, using the Saitzky-Golay filter with order 1 and frame length 31 to filter out noise for each hourglass shaped metastructure. Each setup consists of a base and top aluminum plate (weighing 200 g) under which hourglass samples are sandwiched using fixtures (3D printed PLA white ring) and tightened using 16 N-m torque to ensure the repeatability of each test.

### Analytical evaluation of the hourglass unit cell (equivalent mathematical model)

 The mathematical model of hourglass structure has been treated as a combination of two oppositely oriented coaxial coned-disc spring (Belleville spring) to understand the static and dynamic response characteristics analytically. This facilitates obtaining load-deflection characteristics by combining two non-linear stiffness elements in series (equal stiffness for homogeneous and unequal for non-homogeneous). An equivalent force-deflection relationship has been obtained, which depends on the geometric parameters of constitutive cells. It was observed that the non-linear curves vary in an interesting manner with the geometric parameters. By selecting the values of the parameters judiciously, it is possible to generate the desired non-linear characteristics to obtain customizable stiffness. Angular deflection of the cross-section is assumed to be relatively small so that the cross-section of the hourglass remains undistorted in a deflected position. The vertical loading and base support are uniformly distributed around the respective circumference. The obtained force-deflection relationship for a single lattice dome is as follows:

1 $$\begin{aligned} P=\left( \dfrac{t_c}{l_c}\right) ^{3}\dfrac{E_s}{{\cos }{\theta _c}{\sin }{\theta _c}}\dfrac{\delta }{\left( 1-\nu ^{2}\right) Ma^{3}}\left[ \left( h-\delta \right) \left( h-\dfrac{\delta }{2}\right) t+t^{3}\right] \end{aligned}$$

where *P* is the applied load, $\delta$ is the axial deflection, $E_s$ is the modulus of elasticity, *t* is the radial thickness of hourglass shell, $\nu$ is the Poisson’s ratio, $\theta _c$, $t_c$ and $l_c$ are inclusion cell angle (in case of auxetic structure it is re-entrant angle), cell thickness and cell length respectively, *r* is the ratio of outer *a* and the inner radius *b*, $\alpha$ is the initial cone angle of equivalent coned-disc spring, twice of *h* is the free height of the hourglass structure. The load-deflection relationship is written in the form of cubic polynomial where $K_1$, $K_2$ and $K_3$ are the controlling parameters that govern the type of non-linear stiffness of the hourglass structure

2 $$\begin{aligned} P=K_1\delta +K_2\delta ^{2}+K_3\delta ^{3} \end{aligned}$$

where

3 $$\begin{aligned} \begin{aligned} &K_1=\left( C_1 t h^{2}+C_1 t^{3}\right) , K_2=-\left( \dfrac{3}{2}htC_1\right) , K_3=\left( \dfrac{1}{2}tC_1\right) \\ &C_1= \left( \dfrac{t_c}{l_c}\right) ^{3}\dfrac{E_s \cos \theta _c}{\left( \dfrac{h_c}{l_c}+\sin \theta _c\right) \left( \sin \theta _c\right) ^{2}}\dfrac{1}{\left( 1-\nu ^{2}\right) M a^{2}}\\ & \text {and} \quad \dfrac{1}{M}= \left[ \dfrac{r+1}{r-1}-\dfrac{2}{\log r}\right] \pi \left( \dfrac{r}{r-1}\right) ^{2} \end{aligned} \end{aligned}$$

The combination of d1 and d2 domes is treated as the series of two non-linear springs and used to obtain equivalent relations for homogeneous and non-homogeneous hourglass shaped lattice metastructure (see supplementary information).

Homogeneous hourglass: A series combination of two equal non-linear stiffness having load-deflection characteristics as $P=K_1\delta +K_2\delta ^{2}+K_3\delta ^{3}$. Thus the equivalent load-deflection expression can be obtained using Eqs. (1) and (2) for $P_{eq}$ as

4 $$\begin{aligned} P_{eq}=\dfrac{K_1}{2}\delta _{eq}+\dfrac{K_2}{4}\delta _{eq}^{2}+\dfrac{K_3}{8}\delta _{eq}^{3} \end{aligned}$$

Non-homogeneous hourglass: Treating the structure as a series combination of two different non-linear stiffness with load-deflection characteristics as $P=K_1\delta +K_2\delta ^{2}+K_3\delta ^{3}$ and $P=K_1^{*}\delta +K_2^{*}\delta ^{2}+K_3^{*}\delta ^{3}$, the equivalent expression for $P_{eq}$ may be expressed as

5 $$\begin{aligned} P_{eq}=\dfrac{K_1 K_1^{*}}{K_1+K_1^{*}}\delta _{eq}+\dfrac{K_2K_2^{*}}{\left( \sqrt{K_2}+\sqrt{K_2^{*}}\right) ^{2}}\delta _{eq}^{2}+\dfrac{K_3K_3^{*}}{\left( \root 3 \of {K_3}+\root 3 \of {K_3^{*}}\right) ^{3}}\delta _{eq}^{3} \end{aligned}$$

For initial understanding, a simplification of hourglass shaped lattice metastructure has been applied to establish its basic load-deflection behavior. The Eqs. (4) and (5) are governing load-deflection relations for homogeneous and non-homogeneous class of hourglass metastructure. The coefficients of $\delta _{eq}, \delta _{eq}^{2}, \delta _{eq}^{3}$ are the controlling parameters governing the type of non-linear stiffness of hourglass metastructure.

### Simulations (modal analysis-natural frequency determination)

 The finite element analysis (FEA) simulation has been carried out using Ansys 15.0, as shown in Fig. 5a (see supplementary file for detail). The modal analysis, using mechanical APDL (Ansys parametric design language) solver with tetrahedral mesh (relevance 68, fine sizing) with the number of nodes and elements as 119906 and 5641 respectively for all the six samples. The subspace based algorithm is found suitable to extract modes for complex geometry. The base rib of hourglass is provided a zero displacement constraint to z-axis (hourglass axis) under displacement boundary condition. A comparative study has been carried out for the first ten eigenmodes for all the six samples. The sixth eigenmode is found to be important along the axis of the hourglass structure and directly related to the fundamental axial mode of each sample. It has also been confirmed from the transmissibility experiments.

## Supplementary information

Supplementary material 1

## Data Availability

All relevant data are available from the corresponding author upon reasonable request, and/or are included within the main part and Supplementary Information.
