# Supplementary material for: Exploring the dynamics of hourglass shaped lattice metastructures
Source: Sci Rep. 2020 Dec 1;10:20943. doi: 10.1038/s41598-020-77226-4 (PMC7708529; doi:10.1038/s41598-020-77226-4)
Supplement: Supplementary file 1 — Supplementary material 1 [file 41598_2020_77226_MOESM1_ESM.pdf]

Supplementary Material: Exploring the dynamics of hourglass shaped lattice metastructures

Vivek Gupta[1], Sondipon Adhikari[2], and Bishakh Bhattacharya[1,*]

[1]Department of Mechanical Engineering, Indian Institute of Technology Kanpur, Kanpur, India
[2]College of Engineering, Swansea University, Swansea, UK
[*]Corresponding author: bishakh@iitk.ac.in

Contents

1 Supplementary Discussion **1**
1.1 Introducing the hourglass oscillator . . . 3
2 Supplementary Methods **6**
2.1 Determination of k_{eq} of hourglass shaped unit cell analytically (equivalent mathematical model) . . . 6
2.2 Sample preparation: additive layer manufacturing information . . . 11
2.3 Experiments . . . 11
2.4 Finite element analysis (FEA) simulations . . . 14
2.5 The half-power bandwidth method for damping ζ calculation for each hourglass lattice metastructure . . . 15
2.6 Tensile testing . . . 16
References **17**

1 Supplementary Discussion

The hourglass shaped lattice metastructure consists of combinations of two dome shells d1 and d2 (with and without latticed structures) joined by a smooth spline surface to avoid stress concentration. Two cellular topologies are considered here that are standard in practice based on auxetic (re-entrant angle) and regular honeycomb. The structure is modeled mathematically and the nature of deformation is studied experimentally. We have divided the hourglass metastructure into two classes for a comprehensive study: homogeneous (lattice symmetry between d1 and d2 domes) and non-homogeneous (unsymmetrical lattices between d1 and d2) and the response of different samples have been observed. The mechanical behavior of different lattice structures has been studied corresponding to the double dome formation.
The auxetic lattices are especially favorable to adapt their shape as perfect domes because they exhibit an interesting property of negative Poisson's ratio. The nature of deformation allows them to form a dome-shaped double curvature known as synclastic, which is not found in conventional hexagonal lattices[1–3]. An equivalent analytical model of hourglass shaped metastructure has been formulated by considering it as the combination of two coned-disk springs, which can be further treated as the combination of two nonlinear spring in series. The model introduces the fundamental mechanics of load-deflection behavior under the influence of various topological parameters and different lattice geometry. A good agreement between the experimental and analytical results was achieved.

The static tests were performed in universal testing machine (UTM-20K) under a quasi-static load-deflection relationship. The crosshead speed has been set to 0.5 mm/min (quasi-static strain rate is less than $0.01s^{-1}$) to identify the naturally arising transition points accurately. The deformation mechanism is set at a predetermined strain up to 35% of its initial length H to avoid the effect of the plastic zone. The load-deflection curves in vary linearly up to 3 mm of deflection, and afterward it becomes nonlinear with the 3rd degree of nonlinearity for a particular sample. This behavior is highly customizable by introducing suitable variations in the microstructure and prestressing condition, one can observe a phenomenal change in the nature of deformation as has been elucidated in the later section.

The load-deflection characteristics of hourglass shaped lattice metastructure vary in interesting manner with its geometric parameters. The nature of such variation for homogeneous hourglass (based on auxetic lattice) in terms of the non-dimensional parameters are obtained through this study. Here, P is the load applied along the axis of the sample, δ is the deflection along the same direction, E is the Young's modulus of the constitutive material, the other parameters such as a/b ratio and lattice angle (re-entrant with $-35°$) are kept constant. By selecting the values of the parameters judiciously, it is possible to generate the desired nonlinear characteristics corresponding to different (h/t) ratio. One of the critical observations is the sensitivity of the non-dimensional parameter (h/t), which drastically vary the load-deflection behavior. One can produce a wide 'zero-rate' range by keeping (h/t) close to 3. It was observed that when (h/t) ratio becomes more than 3, the hourglass shaped metastructure can generate negative stiffness, whose magnitude depends upon the lattice topologies (ranging from negative to positive angle). For a lower (h/t) ratio, one can achieve an exponential variation of load-deflection characteristics. Such a variation is desirable while developing isolators for variable loading system[4]. An isolator with constant stiffness cannot function satisfactorily in the entire range of varying inertia (see discussion section). In practice, these characteristics are achieved using a combination of steel leaf and rubber spring, which is a complicated design[5]. Similarly, a suitable generation of nonlinear stiffness could widen the suppression band while designing an effective dynamic vibration neutralizer[6].

The influence of lattice cell angle θ_c on non-dimensional deflection is presented corresponding to the variation in the non-dimensional force. The lattice cell angle varies from negative to positive, transforms the lattice from auxetic (re-entrant angle) to honeycomb. It is observed that the variation of stiffness is nonlinear and significantly high for the honeycomb lattice region while the same stabilizes for the auxetic lattice. It also implies that the snap-through buckling is significantly reduced for re-entrant auxetic lattices in comparison to the honeycomb lattices. The existing literature recognizes that the instability issue of domes due to snap-through buckling can be resolved by introducing the auxetic lattice structure. To understand the dynamics of the hourglass and dome-shaped lattices and their combinations, a detailed experimental study has been conducted to classify the metastructures in terms of vibration response characteristics. We have obtained displacement transmissibility plots for all homogeneous and non-homogeneous samples experimentally.

The experimental results suggest that the natural frequency of developed hourglass metastructure is sensitive to the changes in microstructural lattice topology. The observed peak on the curves at a particular frequency is representative of the corresponding metastructure's natural frequency. The lowest natural frequency is observed for homogeneous auxetic metastructure (67.1 Hz), while the highest corresponds to homogeneous solid shell hourglass (250 Hz). Using the 'half-power bandwidth method' around each peak, damping for each sample has been calculated, as shown in (Table S3). The highest and lowest damping is observed in the case of homogeneous auxetic lattice metastructure with the damping ratio $\zeta = 0.125$ and the non-homogeneous solid shell metastructure with honeycomb lattices with $\zeta = 0.997$ respectively. The damping observed by the other metastructures such as non-homogeneous honeycomb and auxetic lattice with $\zeta = 0.1057$ followed by the non-homogeneous solid shell with auxetic lattice $\zeta = 0.1042$ and homogeneous solid shell (without lattice) with $\zeta = 0.1033$, as shown in Table S3. Thus 21% increment in ζ value is observed in, homogeneous auxetic hourglass metastructure than the homogeneous solid shell metastructure (without lattices). For the same hourglass sample with selected deformation amplitude and frequency range, auxetic lattice structures have higher damping capability than the regular honeycomb lattice. We have obtained a good agreement of transmissibility experiments on laser doppler vibrometer (LDV) with the numerical simulations based on finite element analysis. Modal analysis has been performed for the first ten modes and corresponding modal frequencies have been extracted for each sample. The sixth mode (eigenvalue) is found along the axis of hourglass, which can be directly associated with the natural frequency of each sample.

The transfer function responses for each sample are analyzed under different gains g1(unity), g2, g3, g4 using the FRF data obtained through laser doppler vibrometer up to 500 Hz of the frequency range Fig. S1. The study suggests that the nonlinear effect is insignificant for lower strain values, and all metastructures behave as linear under vibration transmissibility experiment. Notably, the homogeneous auxetic and homogeneous solid shell metastructures are affected significantly at higher gain than the other samples (Fig. S1a).

1.1 Introducing the hourglass oscillator

The hourglass lattice metastructure periodically arranged with n numbers of oscillators may be treated as locally resonant metamaterials. The dispersion analysis based on 1D diatomic system with a second-order lattice based oscillator was carried out mathematically. To tune its bandgap behavior we have introduced a concept of prestressing of hourglass shaped metastructures to get desired nonlinear stiffness response. This tuning of the bandgap is also observed using lattice-dependent stiffness obtained experimentally. An equivalent 1D diatomic locally resonant metamaterial has been considered for initial investigation. The dispersion curves for hourglass based metamaterial with internal resonators having mass ratio $m_r = 0.125$, stiffness $k_{eq} = 50$ N/mm for auxetic and $k_{eq} = 266$ N/mm for honeycomb oscillator, has been obtained. It is found under various gains that the transmissibility response does not vary significantly for small deflections (Fig. S1a). Hence, to avoid further complexities, we have considered linear stiffness obtained experimentally. A solution of the dispersion relationship for harmonic wave motion i.e., for assigned values of frequency provides the real and imaginary parts of the wavenumber ν. The $\Omega_r = \omega_r/\omega_0$ and $\Omega = \omega/\omega_0$ can be tuned to obtain attenuation over the desired band, without any constraints imposed by wavelength[7]. For low-frequency attenuation, auxetic lattice-based hourglass and for a higher range of frequency, honeycomb lattice-based hourglass is found to be effective. However, an optimized nonlinear stiffness of hourglass metastructure can be obtained by tuning its (h/t) ratio and θ_c to widen the bandgap. Such metastructure may work as a building block of damped nonlinear phononic materials, which can have the potential applications to enable tunable filters, waveguides, resonators, frequency isolators, and acoustic diodes[7].

The dispersion curves for hourglass lattice metastructure with internal resonators have been obtained for $m_r = 0.125$, k_{eq} =50 N/mm(for auxetic), $k_{eq} = 266$ N/mm(for honeycomb) lattices based metastructure. The k_{eq} values obtained experimentally. Equations of motion for 1D periodic spring mass chain with internal oscillator can be written as:

$$\begin{aligned}&(-\omega^2 m_R + 2k_{eq})u_n - k_{eq}(u_{n-1} + u_{n+1}) - k_r(u_{r_n} - u_n) = 0\\&(-\omega^2 m_r + k_r)u_{r_n} - k_r u_n = 0\end{aligned} \tag{S1}$$

Where, m_R, k_{eq}, respectively are the mass of metallic ring and stiffness of hourglass oscillator. m_r, k_r, respectively are the resonating mass and its connected spring stiffness. where ω is the temporal frequency of harmonic motion, u_n displacement of nth mass, u_{n+1} and u_{n-1} contains the neighboring degree of freedom (DOF) of the nth unit cell of the lattice. Equation (S1) can be written in condensing form in resonators degree of freedom, gives

$$(-\omega^2 m_R + 2k_{eq} - \frac{k_r^2}{k_r - \omega^2 m_r} + k_r)u_n - k(u_{n-1} + u_{n+1}) = 0 \tag{S2}$$

Application of Bloch's theorem subjected to the periodic condition (all homogeneous hourglass) and some manipulations lead to the following dispersion relationship.[7]

$$\hat{u_n}(\omega) = \tilde{u}(\nu(\omega))e^{\nu n} \tag{S3}$$

Where, ν is the wave number

$$2(1 - \cos(\nu)) - \Omega^2(1 + \frac{\bar{m_r}}{1 - \Omega^2/\Omega^2}) = 0 \tag{S4}$$

Where $\Omega = \omega/\omega_R$ and $\Omega_r = \omega_r/\omega_R$, while $\bar{m_r} = m_r/m_R$. The dispersion curves for hourglass lattice metastructure with internal resonators have been obtained for $m_r = 0.125$, $k_{eq} = 50$ N/mm (for auxetic), $k_{eq} = 266$ N/mm (for honeycomb) lattices based metastructure. Solution of the dispersion expression Eq. (S4) for harmonic wave motion, for assigned values of frequency provides the real and imaginary parts of ν. Internal resonator splits the dispersion curves into two branches, between this a bandgap is observed.

The undeformed height H of all samples is 48 mm. From the amplitude gain results, it is evident that the nonlinear effect is insignificant at lower strain values. It may be substantiated from the analytical results obtained for different

undeformed heights H, moreover, for height nearly 12 mm produces a wider-zero rate range with zero stiffness showing significant nonlinear effects at lower strain values. This range of height is suitable for designing nonlinear oscillators. Coupling with piezoelectric bimorph offers an exciting aspect of switching the stiffness zones by pre-stressing the hourglass metastructure. If n number of hourglass oscillators are treated as nonlinear phononic material with resonating lumped mass using tunable stiffness, it can produce a wider attenuation zone[7]. The presence of nonlinear terms in the equation of motion does not allow Bloch's theorem to be invoked in the usual manner, this may overcome by perturbation approach[8].

Notable manifestation of engineering science was involved in the construction of roman domes/arch architectures[9,10]. The central idea that makes the awe-inspiring structures possible is based on simple Newtonian physics. The interplay of gravitational force and the equilibrium forces cause compression on particular segments. On the other hand, an arch/dome lets us redirect forces in a way that pushes towards its center of gravity for discreet blocks or lattices. The lattices are introduced in a dome shape to enhance their functionality due to compression forces. The hourglass metastructure, which takes advantage of the combination of two oppositely oriented coaxial domes, provides an opportunity of higher customizability and the ability to tailor the dynamic response. Different nonlinear stiffness profiles have been observed which are sensitive to its non-dimensional h/t ratio and lattice cell angle θ_c. The lattice dependent natural frequencies have been obtained experimentally. The nonlinear load-deflection responses for a small value of the undeformed height of hourglass H is exponential which is important for the applications where the natural frequency of the system is required to be constant over a wide range of applied load[5,6]. For undeformed height, $H = 12$ one can generate a wide-zero rate range where the zero value of stiffness is observed. The magnitude of load-bearing capacity can be tuned by altering the lattice cell angle from (-ve) re-entrant to honeycomb. It is observed that the natural frequency and stiffness of auxetic lattice based hourglass are less than the honeycomb lattice structure. By providing suitable variations on the re-entrant angle, one can obtain attenuation over the desired band without any constraint imposed by the wavelength. In actual practice, low-frequency attenuation requires low stiffness always poses a challenge to the researchers[7].

In summary, six lattice-based hourglass metastructures have been proposed, analytically modelled and analyzed through experimental and numerical studies to investigate their mechanical and structural properties. The realization of their static and dynamic responses is obtained through the quasi-static compression and transmissibility tests. A lattice-based comparative study has been conducted by classifying the homogeneous and non-homogeneous category of 3D printed hourglass samples. The governing load-deflection relationship has been obtained analytically, which reports the nonlinear stiffness variation of hourglass metastructure and its sensitivity to lattice topologies with non-dimensional parameters. The transmissibility tests of all six samples facilitate the lattice-based comparison to obtain the different stiffness profiles and lattice dependent natural frequencies. The auxetic based hourglass samples and their combinations are observed to be less stiff than the honeycomb lattice-based, which shifts its natural frequency to the higher side. The damping values for each sample have been evaluated experimentally. Homogeneous class of auxetic based hourglass structure offers the highest damping compare to other samples. The superior functionality of auxetic lattices has been observed by incorporating them into the dome shape of the hourglass structure, making re-entrant lattice suitable for better energy absorption than the other combinations. The first ten modal frequencies and mode shapes were extracted through the numerical simulations. The sixth mode shape is observed along the longitudinal axis of the hourglass metastructure. A close agreement is obtained through the experimental validations. The emergence of lattice based different nonlinear stiffness profiles and damping values have been estimated through the experimental, analytical, and numerical simulation for a novel hourglass shape metastructure. Further experimental studies investigating the different lattice functionality with a variety of dome shapes may be required to understand the critical geometrical shape responsible for its mechanics. Combining these experiments with analytical and numerical studies will allow a structural picture of a customizable mechanical response to be model. From this foundation, one can envisage being able to design an hourglass based lattice metastructure as a building block of tunable metamaterial and a novel unit cell for vibration isolation bed.

We propose the first step toward contemplating this hourglass shape in the lattice based metastructure design. Based on the observations from this study, the lattice functionality is dependent on the shape of the host structure, and superior customizability of hourglass shape is proven to obtain the desired mechanical response.

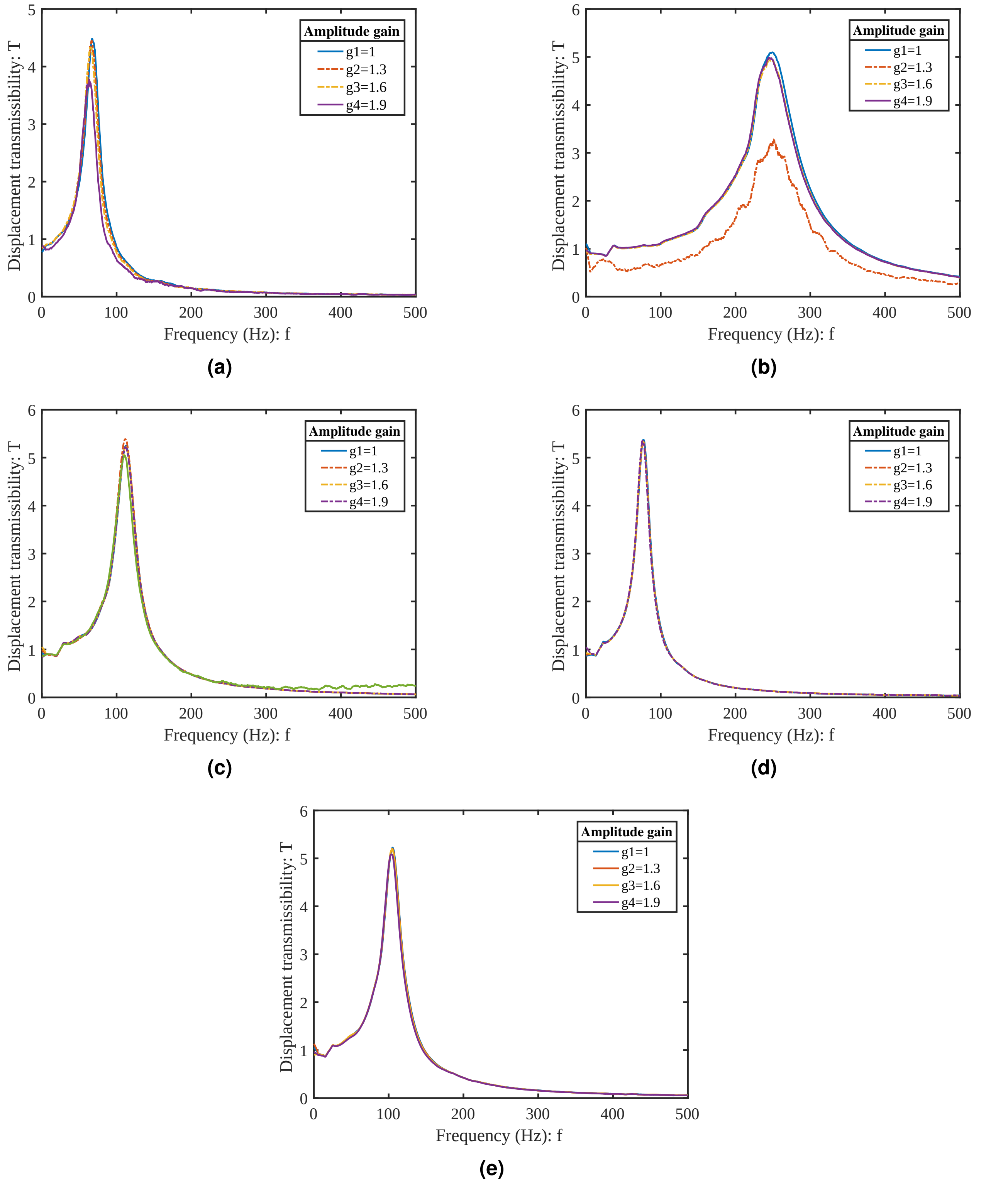

Figure S1. The displacement transmissibility plots (under different gain g1(unity), g2, g3, g4) obtained experimentally (tested in LDV) using the data of frequency response function (FRF). All 3D printed hourglass metastructures subjected to base excitation with pseudo-random signal (1600 FFT lines) under the frequency range 0-500Hz. Homogeneous hourglass metastructure: **(a)** auxetic lattice with gain, **(b)** solid shell, non-homogeneous: **(c)** non-homogeneous hourglass solid shell with auxetic lattices, **(d)** honeycomb with auxetic lattices, **(e)** solid shell with honeycomb lattices.

2 Supplementary Methods

2.1 Determination of k_{eq} of hourglass shaped unit cell analytically (equivalent mathematical model)

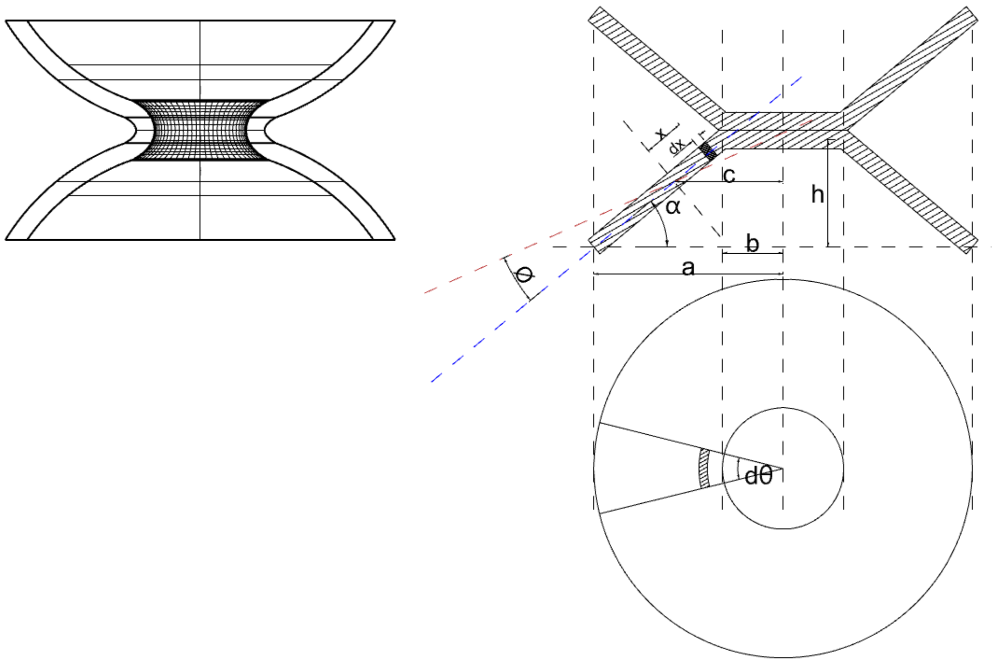

Figure S2. A simplified geometrical model of hourglass shaped lattice metastructure considered here as a series combination of co-axially inverted coned disk springs.

An equivalent analytical model of the hourglass structure has been derived by treating it as a combination of two oppositely oriented coaxial conical disks. The method used here follows in general that used by Timoshenko[11–13] by assuming negligible radial stresses and the cross-section of the disk does not distort, rather than merely rotates about a neutral point O, shown in Fig. S2. To obtain the load-deflection relationship, consider a sector $d\theta$ of the conical disk and dx is in x distance from O. When the disk is deflected through an angle ϕ, due to δ deflection this strip moves to position indicated by a dashed line. Therefore, the general strain may be analyzed as the resultant of radial displacement of dr and a rotation of ϕ. The first of these produces a uniform strain throughout the disk's thickness, neglecting small variation in distance to the center of the disk at different points of section. The second causes tangential bending strain, which is zero in the neutral surface and maximum at the upper and lower surfaces. The tangential stresses produced by these two components of the strain (M_{r1} and M_{r2}) causes a radial moment M_R about point O which resists the external moment created by applied forces. This facilitates to obtain load-deflection characteristics by combining two nonlinear stiffness in series (equal stiffness for homogeneous and unequal for non-homogeneous). An equivalent force-deflection relationship has been obtained which depends on the geometric parameters of constitutive cells. Assuming the cross-section of angular deflection is relatively small, it remains undistorted in a deflected position. The vertical loading and base support are uniformly distributed around the respective circumference.

Where, P is the applied load, δ is the axial deflection, E_s is the modulus of elasticity, t is the radial thickness of hourglass shell, θ_c, t_c and l_c are included cell angle (in case of auxetic it is re-entrant angle), cell thickness and cell length respectively, r is the ratio of outer a and inner b radius, α is the initial cone angle of equivalent coned disk, twice of h is the free height (undeformed) of hourglass structure. To derive force-deflection relation, first calculate tangential stress due to radial displacement: The length of section dx before deflection l_1, after deflection l_2 and the

change of length $l_2 - l_1$ in equation (S5).

$$\begin{aligned} l_1 &= d\theta(c - x\cos\alpha) \\ l_2 &= d\theta[c - x\cos(\alpha - \phi)] \\ l_2 - l_1 &= d\theta[-x\cos\alpha(1-\cos\phi) + x\sin\alpha\sin\phi] \end{aligned} \tag{S5}$$

Assuming the angle α is small we have

$$\cos\alpha \approx 1, \quad \sin\alpha \approx \alpha, \quad \text{and} \quad 1 - \cos\phi \approx 2\sin^2\phi = \frac{\phi^2}{2}$$

Change in length can be written as

$$l_1 - l_2 = d\theta x\phi\,(\alpha - \phi/2)$$

Now, the tangential strain ε_θ written as approximately

$$\varepsilon_\theta = \frac{l_1 - l_2}{l_1} = \frac{x\phi\,(\alpha - \phi/2)}{(c - x)} \tag{S6}$$

The tangential stress $\sigma_{\theta'}$ defined as

$$\sigma_{\theta'} = \frac{E_{eq}}{1-\mu^2}\,(\varepsilon_2 + \mu\varepsilon_1)$$

Since, the radial stresses are negligible, we can write

$$\sigma_{\theta'} = \frac{E_{eq}\varepsilon_2}{1-\mu^2} = \frac{E_{eq}x\phi\,(\alpha - \phi/2)}{(c-x)\,(1-\mu^2)} \tag{S7}$$

Now, the radial moment dM_{r1} due to tangential forces in the section about point O is

$$dM_{r1} = \sigma_{\theta'}\,t\,dx\,d\theta x\sin(\alpha - \phi)$$

For small angles, $\sin(\alpha - \phi) \approx (\alpha - \phi)$, substituting Eq. (S7) to the radial moment expression dM_{r1} gives

$$dM_{r1} = \frac{E_{eq}x\phi\,(\alpha - \phi/2)}{(c-x)\,(1-\mu^2)}\,t\,dx\,d\theta\,x\,(\alpha - \phi)$$

and then integrating from $x = c - a$ to $x = c - b$, to get the internal moment of the sector about O

$$M_{r1} = \frac{E_{eq}\phi\,(\alpha - \phi/2)}{(1-\mu^2)}\,t\,d\theta\,(\alpha - \phi)\int_{c-a}^{c-b}\frac{x\,dx}{(c-x)}$$

Radial moment of the tangential forces about point O

$$M_{r1} = \frac{E_{eq}\phi\,(\alpha - \phi/2)}{(1-\mu^2)}\,t\,d\theta\,(\alpha - \phi)\left[\frac{1}{2}\left(a^2 - b^2\right) - 2c\,(a-b) + c^2\log a/b\right] \tag{S8}$$

Now we proceed to the calculation of the other part of the total resisting moment, that is, the tangential stress due to the change of curvature. Hence, bending strain is developed by the moment. Where κ_1 and κ_2 are the change of radial and tangential curvature defines as

$$M = D(\kappa_2 + \sigma\kappa_1)$$

As per the standard sign convention, M will be positive for decreasing the conical shell height. Bending in the radial section being neglected, hence differential bending moment for a section of length dx

$$dM = D\kappa_2 dx = \frac{Et^3}{12(1-\sigma^2)}\kappa_2 dx$$

For unloaded conical disk, tangential curvature can be written as[12]

$$\frac{\sin\alpha}{c-x}$$

and that of the deflected one

$$\frac{\sin(\alpha-\phi)}{c-x}$$

Hence, the change of curvature can be written as

$$\kappa_2 = \frac{\sin(\alpha)-\sin(\alpha-\phi)}{c-x}$$

Substituting the small angle $\sin\alpha \approx \alpha, \sin(\alpha-\phi) \approx (\alpha-\phi)$, and then using equations with in the text yields expression for the differential moment dM as

$$dM = \frac{E_{eq}t^3\phi\, dx}{12(1-\sigma^2)(c-x)}$$

The tangential stress $\sigma_{\theta''}$ at the surface due to change of curvature may be written as using dM as

$$\sigma_{\theta''} = \frac{6dM}{t^2dx} = \frac{E_{eq}\phi}{(1-\sigma^2)(c-x)}\frac{t}{2}$$

$$\sigma_{\theta''} = \frac{E_{eq}\phi}{(1-\sigma^2)(c-x)}y$$

The component of dM in radial direction may be written as

$$dM_{r2} = 2dM\frac{d\theta}{2}$$

Integrating dM_{r2} for the whole sector from $x = c-a$ to $x = c-b$, becomes

$$M_{r2} = \frac{E_{eq}t^3\phi d\theta}{12(1-\mu^2)}\int_{c-a}^{c-b}\frac{dx}{c-x} = \frac{Et^3\phi d\theta}{12(1-\mu^2)}\log\frac{a}{b} \tag{S9}$$

Total radial moment is obtained by summing up the radial moments due to radial displacement Eq. (S8) and due to change of curvature Eq. (S9). The total radial moment signifies net resisting moment developed against the external applied load.

$$M_R = M_{r1} + M_{r2} = \frac{E\phi d\theta}{(1-\mu^2)}\left[\left(\frac{1}{2}\left(a^2-b^2\right) - 2c\left(a-b\right) + c^2\log\frac{a}{b}\right)(\alpha-\phi)\left(\alpha-\frac{\phi}{2}\right)t + \frac{t^3}{12}\log\frac{a}{b}\right] \tag{S10}$$

The external moment on sector $d\theta$ due to applied load along the hourglass axis equals

$$\frac{P(a-b)d\theta}{2\pi}$$

This must be equal to the internal resisting moment, hence

$$P = \frac{2\pi M_R}{(a-b)\,d\theta}$$

The following substitution have been used to get the final expression, defined as

$$\alpha = \frac{h}{a-b};\ \phi = \frac{\delta}{a-b};\ r = \frac{a}{b}$$

Assuming the outer and inner planes of the spherical shell are stress-free, thus, radial and shear stress values may be zero on each face. Because the region is thin in the radial direction, there can be a little variation in these stresses components through the thickness, and thus they will be approximately zero throughout the entire domain. For the initial investigation, taking a thin region along the radial direction and have a little variation of stresses with radius. These arguments can be summarized as plane stress state under no body forces and surface tractions along the radial direction[11,14]. Hence, incorporating the in-plane modulus of elasticity of hexagonal lattice. Now, introducing the E_{eq}, which is applicable for re-entrant $(-\theta_c)$ and $(+\theta_c)$ regular honeycomb angle written in terms of cellular topological parameters. Where, E_{x1} and E_{x2} are the modulus of elasticity in $x1$ and $x2$ direction, θ_c, t_c, $h_c$and l_c are included cell angle (in case of auxetic it is re-entrant angle), cell thickness, height and cell beam length respectively, E is Young's modulus of elasticity of constitutive material[15], can be written as

$$\begin{aligned} E_{x1} &= \left(\frac{t_c}{l_c}\right)^3 \frac{\cos\theta_c}{\left(\frac{h_c}{l_c}+\sin\theta_c\right)\sin^2\theta_c} E_s \\ \nu_{12} &= -\frac{\varepsilon_2}{\varepsilon_1} = \frac{\cos\theta_c^2}{(\frac{h_c}{l_c}+\sin\theta_c)\sin\theta_c} \end{aligned} \tag{S11}$$

Note that, for regular hexagonal lattices the reciprocal theorem is applies. Hence, Poisson's ration follows rec

$$E_{x2} = \left(\frac{t_c}{l_c}\right)^3 \frac{\left(\frac{h_c}{l_c}+\sin\theta_c\right)}{\cos^3\theta_c} E_s$$

Equating net internal resisting moment M_R, using Eq. (S10) to external applied moment due to load P. The final expression for load-deflection can be obtained using Eqs. (S11) for a lattice shaped dome, yielding the expression for P as

$$P = \left(\frac{t_c}{l_c}\right)^3 \frac{E_s}{\cos\theta_c\sin\theta_c}\frac{\delta}{(1-\nu^2)Ma^3}\left[(h-\delta)\left(h-\frac{\delta}{2}\right)t+t^3\right] \tag{S12}$$

This Eq. (S12) is the expression for one metastructure dome i.e. for d1 dome. To get combined load-deflection relation for d1 and d2 domes. Here, we have compare it with a series combination of two nonlinear springs. Which have been derived from Eq. (S15) to Eq. (S18). Here, load P can be written in a simplified polynomial expression in term of δ can be written as

$$P = K_1\delta + K_2\delta^2 + K_3\delta^3 \tag{S13}$$

Here, load-deflection relationship is written in the form of cubic polynomial where K_1, K_2 and K_3 are the controlling

parameters which governs the type of nonlinear stiffness of the hourglass structure defined as

$$
\begin{aligned}
K_1 &= \left(C_1 t h^2 + C_1 t^3\right), K_2 = -\left(\frac{3}{2} h t C_1\right), K_3 = \left(\frac{1}{2} t C_1\right) \\
C_1 &= \left(\frac{t_c}{l_c}\right)^3 \frac{E_s \cos\theta_c}{\left(\frac{h_c}{l_c} + \sin\theta_c\right)(\sin\theta_c)^2} \frac{1}{(1-\nu^2) M a^2} \\
\text{and} \quad \frac{1}{M} &= \left[\frac{r+1}{r-1} - \frac{2}{\log r}\right] \pi \left(\frac{r}{r-1}\right)^2
\end{aligned}
\tag{S14}
$$

Combination of d1 and d2 domes treated as the series combination of two nonlinear springs

Formulating equivalent relations for homogeneous and non-homogeneous hourglass shaped lattice metastructure by considering it as **combination of two nonlinear spring**. If two purely nonlinear spring arranged in series. The spring constants are C_1 and C_2. Thus, the force characteristics $P = C_1 x^N$ and $P = C_2 x^N$, is satisfied. Expressing x and y then summing them up in $z = x + y$, one can drive

$$
P = \frac{1}{\left(\left(\frac{1}{C_1}\right)^{\frac{1}{2n+1}} + \left(\frac{1}{C_2}\right)^{\frac{1}{2n+1}}\right)^{2n+1}} Z^{2n+1} \qquad \text{If N is odd power; } N = 2n+1 \tag{S15}
$$

$$
P = \frac{1}{\left(\left(\frac{1}{C_1}\right)^{\frac{1}{2n}} + \left(\frac{1}{C_2}\right)^{\frac{1}{2n}}\right)^{2n}} Z^{2n} \qquad \text{If N is even power; } N = 2n \tag{S16}
$$

Homogeneous hourglass: Treating structure as a series combination of two equal nonlinear spring having load-deflection characteristics as $P = K_1\delta + K_2\delta^2 + K_3\delta^3$. The equivalent load deflection characteristics can be obtained using Eqs. (S15) and (S16) yielding the expression for P_{eq} as

$$
P_{eq} = \frac{K_1}{2}\delta_{eq} + \frac{K_2}{4}\delta_{eq}^2 + \frac{K_3}{8}\delta_{eq}^3 \tag{S17}
$$

Non-homogeneous hourglass: Here, treating structure as a series combination of two different nonlinear spring having load-deflection characteristics as $P = K_1\delta + K_2\delta^2 + K_3\delta^3$ and $P = K_1^*\delta + K_2^*\delta^2 + K_3^*\delta^3$. The equivalent load deflection characteristics can be obtained using Eqs. (S15) and (S16) yields the expression for P_{eq} as

$$
P_{eq} = \frac{K_1 K_1^*}{K_1 + K_1^*}\delta_{eq} + \frac{K_2 K_2^*}{\left(\sqrt{K_2} + \sqrt{K_2^*}\right)^2}\delta_{eq}^2 + \frac{K_3 K_3^*}{\left(\sqrt[3]{K_3} + \sqrt[3]{K_3^*}\right)^3}\delta_{eq}^3 \tag{S18}
$$

For initial understanding, a simplification of hourglass shaped lattice metastructure has been done to establish its basic load-deflection behavior. The Eqs. (S17) and (S18) are governing load-deflection relations for homogeneous and non-homogeneous class of hourglass metastructure respectively. The coefficients of $\delta_{eq}, \delta_{eq}^2, \delta_{eq}^3$ are the controlling parameters governing the type of nonlinear stiffness of hourglass metastructure.

2.2 Sample preparation: additive layer manufacturing information

Materials A flexible 3D printing material PCTPE (plasticized copolyamide thermoplastic elastomer) provided by Taulman 3D (see Table S2) is used to print hourglass metastructure as an experimental sample, and high strength PLA (polylactic acid thermoplastic) is used for its fixture. The basic properties of PCTPE material are characterized by a Young's modulus of $E = 75$ Mpa, Poisson's ratio $\nu = 0.28$, and density $\rho = 0.96$ gm/cm^3. The flexibility of material ensures, all the lattices to be functional under the applied load. An aluminum metal plate with density $\rho = 2.7$ gm/cm^3 , radius 50 mm and thickness 8 mm were used as dead weight and base plate for each test (see in Fig. S7).

Fabrication using 3D printing. The modeling of homogeneous (d1 and d2 domes with lattice symmetry) and non-homogeneous (d1 and d2 domes with unsymmetrical lattices) hourglass samples have been fabricated using Ultimaker 3.0 Extended multi-material 3D printer. The developed metastructure sample is printed as a whole body with d1 and d2 domes joined by a smooth spline surface to avoid stress concentration. Each dome is constituted with standard lattices i.e., auxetic, regular honeycomb, and solid shell type. The sphere radius of d1 and d2 domes are 50 mm, dome height (h) is 24 mm (makes the undeformed height of hourglass $H = 48$ mm), radial thickness 4 mm with base radius is 42 mm. Each lattice beam has a thickness of 1 mm. The 3D printing specification with slicing 0.15 mm layer thickness resolution, 100% infill density with triangular infill pattern, and necessary rectilinear and zig-zag supports (minimum 45° overhang angle) were used to prevent the dome from collapsing (see Table S1), as shown in Fig. S3. The support pattern used for honeycomb lattices were gyroid type, which is found easy to remove and for others zig-zag type found suitable. The detailed parameters are shown in Table S1. Within the limitation of 3D printing technology, the layer orientation is found to influence the mechanical properties of the material. Therefore, all the samples are printed along with the same orientation on the build platform.

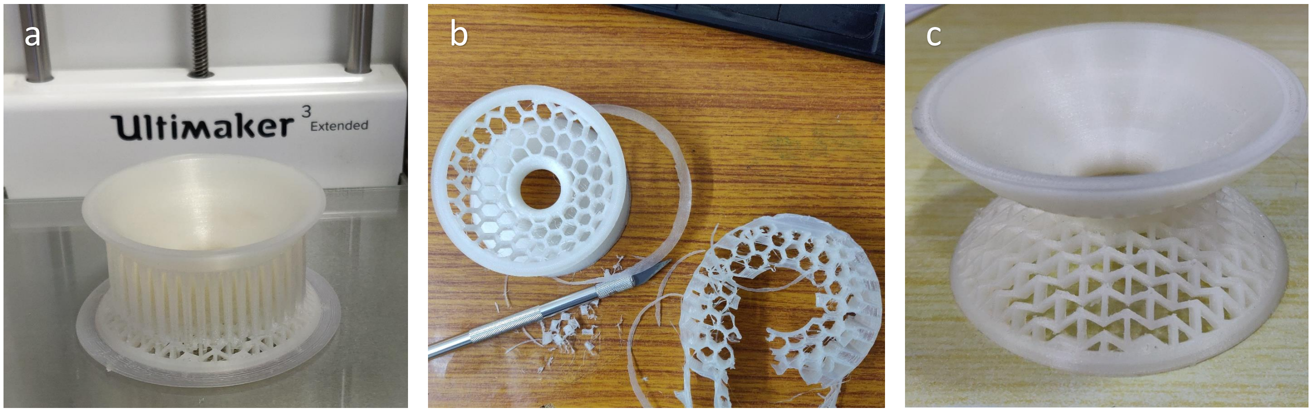

Figure S3. 3D printing: **(a)** Finished 3D printed hourglass shaped metastrcuture (auxetic lattice based) with brim and supports. **(b)** Removal of all supports with the help of precision knife, **(c)** a well finished non-homogeneous hourglass shaped lattice metastructure sample with all functionally active lattices.

2.3 Experiments

Determination of k_{eq} of hourglass shaped unit cell experimentally (static and dynamic tests).

The computer-aided design for all six hourglass shaped metastructure unit cell has been developed in solid modeling software with the dimensions: the sphere radius of d1 and d2 domes are 50 mm, dome height (h) is 24 mm (makes the undeformed height of hourglass $H = 48$ mm), radial thickness 4 mm with base radius is 42 mm. Each lattice beam has a thickness of 1 mm. Furthermore, the stereolithography file (STL) is used for 3D printing and the initial graphics exchange specification file (IGES) imported to finite element (FE) based simulation software Ansys.

Static testing. The measurement of load-deflection characteristics of hourglass shaped (re-entrant auxetic) 3D printed unit cell has been carried out through the loading-unloading tests in universal testing machine (Instron UTM-1195 with 2KN load cell), as shown in Fig. S4. The cross-head speed of 0.5 mm/min (quasi-static) has been set to get exact naturally arising transition points. Furthermore, the data points have been processed and plotted in Matlab R2019b to identify the nonlinear stiffness behavior of the sample. The experimental results have been analyzed and approximated with a third and fourth-degree polynomial to estimate its experimental nonlinear stiffness from load-deflection characteristics.

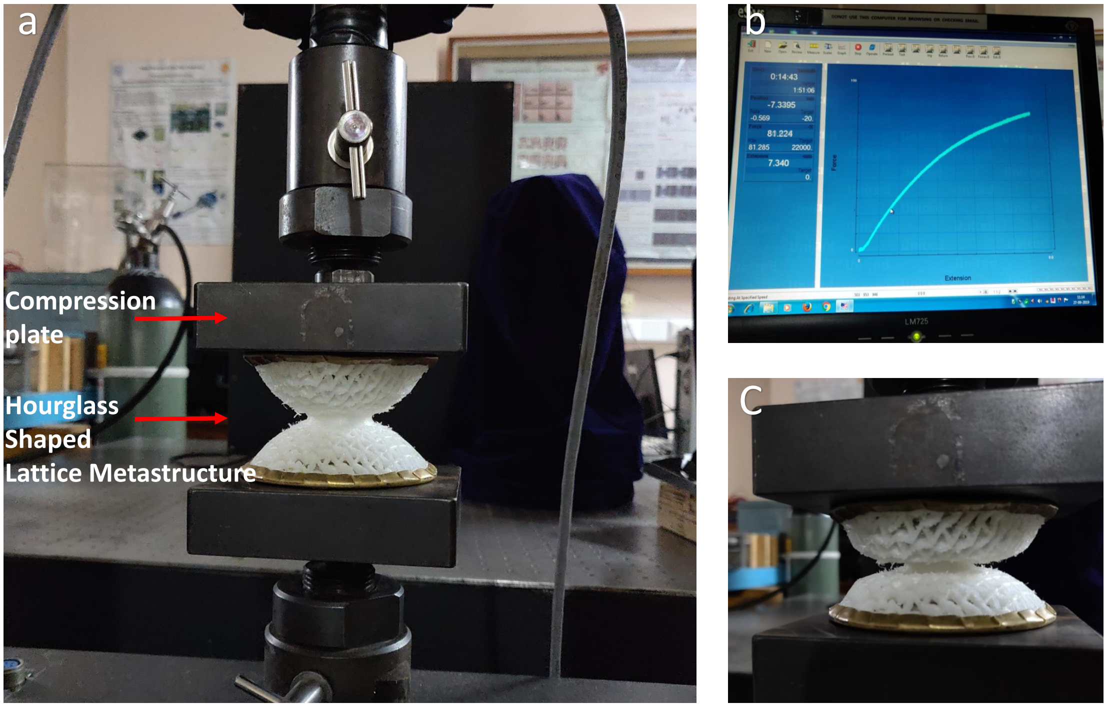

Figure S4. Experimental setup for static testing (universal testing machine UTM-20K), a homogeneous auxetic lattice metastructure has been considered for obtaining load-deflection relationship under quasi-static strain rate with crosshead speed 0.5 mm/min (less than $0.01 s^{-1}$) and up to 35% compression from the initial height of the sample.

Dynamic testing. The experimental dynamic responses of hourglass shaped metastructure are obtained using non-contact vibration measurement techniques. A comparative study of all six samples has been performed using 3D laser doppler vibrometer (LDV) from Polytec, mounted on a tripod, is used to measure the velocity along the beam, and the NI-DAC systems are used for data acquisition and signal processing (Fig. S5). A retro-reflective tape is applied to the top and bottom plate (under which hourglass samples are sandwiched), enhancing its ability to reflect the incident laser beam. The displacement transmissibility tests are performed using base excitation using pseudo-random signal (1600 FFT lines) and sine sweep, performed by LDS-Electrodynamic shaker (V780). The frequency response function data (FRF) up to 500 Hz were analyzed under different gains, using the Saitzky-Golay filter with order 1 and frame length 31 to filter out noise for each hourglass shaped metastructure. Each setup consists of a base and top aluminum plate (weighing 200 gms) under which hourglass samples are sandwiched using fixtures (3D printed PLA white ring) and tightened with bolts with fix 16 N-m torque to ensure the repeatability of each test.

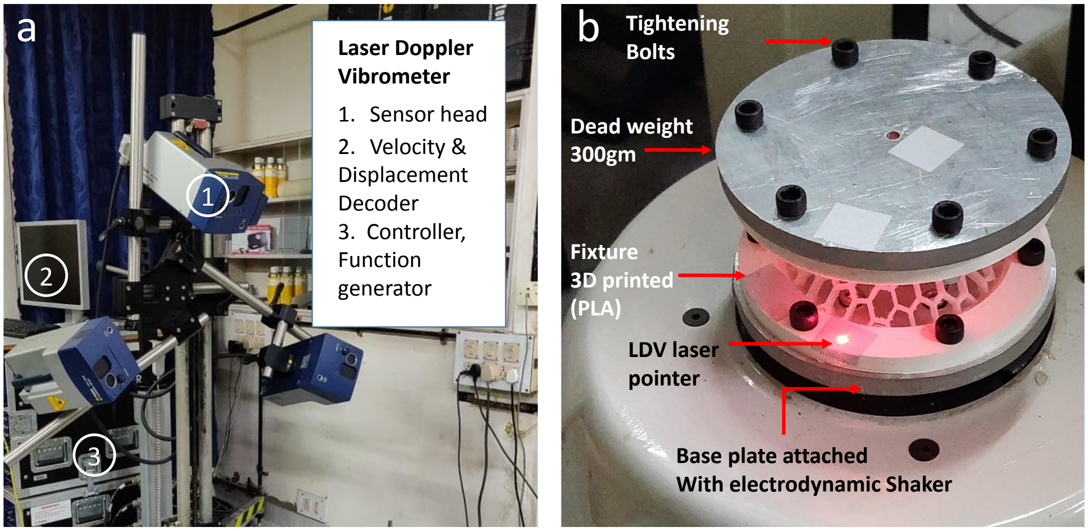

Figure S5. Experimental setup: **(a)** Elements of laser doppler vibrometer (LDV), **(b)** all attachments: hourglass honeycomb lattice metastructure, the white ring is the fixture (3D printed) to fix hourglass with the metal plate (top and bottom), the lower ring is the part of the base plate attached with the shaker, and the upper one is the part of deadweight (total weight 300gms plate+fixture+bolts).

S.N	3D printing specifications	Values
1	Material	PCTPE (hourglass) PLA (fixtures) (2.85 mm nominal dia)
2	Layer height thickness	0.15 mm
3	Infill density	100 %
4	Infill pattern	Triangular
5	Printing temperature	235°C
6	Build plate temperature	85°C
7	Print speed	60 mm/s
8	Support placement	Support overhang angle 45°
9	Support pattern	Zig-zag (auxetic and solid shell), gyroid (honeycomb lattice)
10	Support density	15 %
11	Support line distance, infill layer support line distance	2.3mm (both)
12	Build plate adhesion type	Brim, raft

Table S1. A detailed 3D printing specifications used while developing the hourglass samples.

S.N	Mechanical properties	Values
1	Young's modulus of elasticity (E)	PCTPE: 75 mpa (hourglass)<br>PLA (fixtures): 15 GPa
2	Poisson's ratio	0.28
3	Density	PCTPE: $0.96 gm/cm^3$<br>PLA: $1.2 gm/cm^3$

Table S2. Mechanical properties of the 3D printing materials.

2.4 Finite element analysis (FEA) simulations

Simulations (Modal Analysis-natural frequency determination):

The finite element analysis (FEA) based simulation has been carried out using Ansys 15.0 as shown in Fig. S6. The modal analysis, using mechanical APDL (Ansys parametric design language) solver with tetrahedral mesh (relevance 68, fine sizing) with the number of nodes and elements, are 119906 and 5641 used for all the six samples. The subspace based algorithms is found suitable to extract modes for complex geometry. The base rib of hourglass is provided a zero displacement constraint to z-axis (hourglass axis) under displacement boundary condition. A comparative study has been carried out for the first ten eigenmodes for all the six samples. The sixth eigenmode is found to be important along the axis of the hourglass structure and directly related to the fundamental axial mode of each sample. It has also been confirmed from the transmissibility experiments.

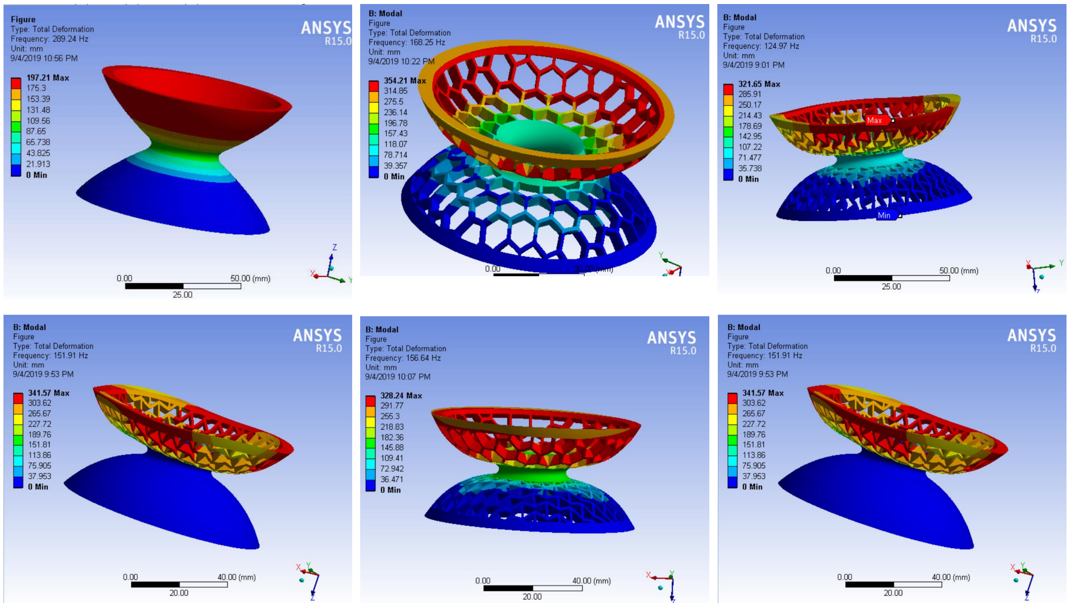

Figure S6. FEA simulation results of modal analysis, homogeneous and non-homogeneous hourglass shaped lattice metastructure deformation response under the sixth mode along the hourglass axis. The base rib of hourglass is provided a zero displacement constraint to z-axis (hourglass axis) under displacement boundary condition. The lowest natural frequency is observed for homogeneous auxetic metastructure while the highest corresponds to homogeneous solid shell hourglass.

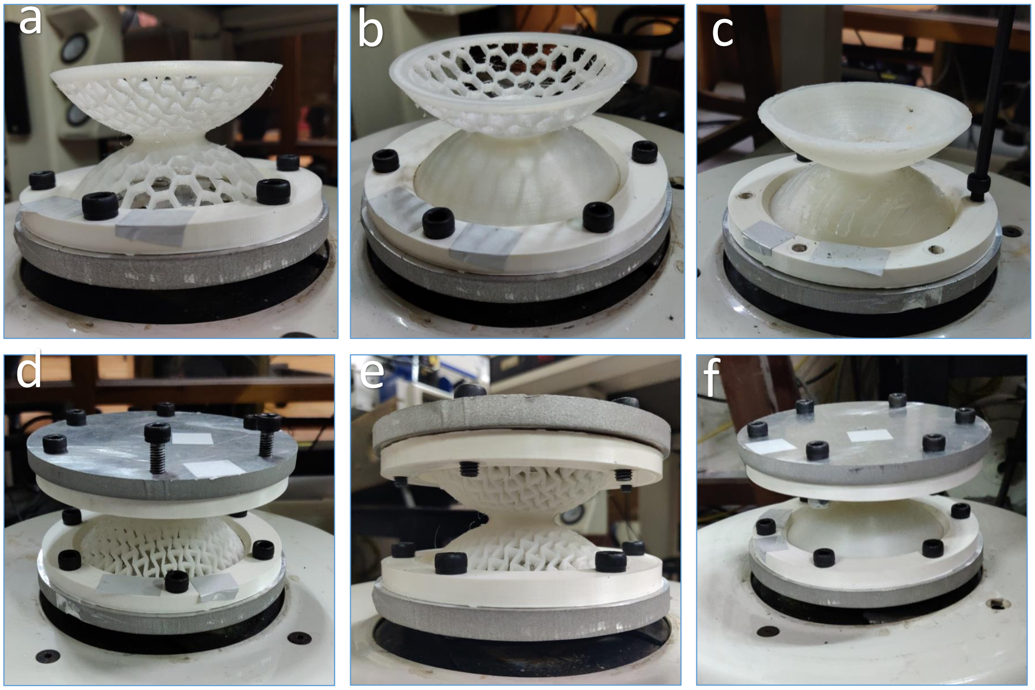

Figure S7. Hourglass lattice metastructure samples, fixed adequately with the base plate attached with electrodynamic LDS shaker and deadweight tightened with the help of fixtures. **(a), (b), (c)** Non-homgeneous samples and **(d), (e), (f)** homogeneous samples. A retro-reflective tape is applied to the top and bottom plate (under which hourglass samples are sandwiched), enhancing its ability to reflect the incident laser beam.

2.5 The half-power bandwidth method for damping ζ calculation for each hourglass lattice metastructure

Damping in mechanical systems may be represented in numerous formats. The most common forms are Q and ζ , where Q is the amplification or quality factor ζ is the viscous damping ratio or fraction of critical damping. Using the 'half power bandwidth method' around each peaks, damping for each sample has been calculated and shown in Table S3. The highest damping is observed in the case of homogeneous auxetic lattice metastructure with the damping ratio $\zeta = 0.125$, followed by non-homogeneous metastructure based on honeycomb and auxetic lattices with $\zeta = 0.1057$, non-homogeneous metastructure based on solid shell and auxetic lattices with $\zeta = 0.1042$, homogeneous metastructure of solid shell without lattices with $\zeta = 0.1033$, and non-homogeneous metastructure based on solid shell and honeycomb lattices with $\zeta = 0.0997$. The 21% increment in ζ value is observed in auxetic lattice based hourglass metastructure than the solid shell hourglass and approx 30% increment in damping is observed than the honeycomb lattice.
These two parameters are related by the formula:

$$Q = \frac{1}{2\zeta} \tag{S19}$$

The Q value is equal to the peak transfer function magnitude for a single-degree-off freedom subjected to base excitation at its natural frequency. Here we define the quality factor or Q factor, which characterizes the system's bandwidth relative to the resonant frequency. When the Q factor is low, the resonant peak becomes less significant, and the measurement of finding the change in the resonant frequency becomes difficult. The Q factor is also defined

as the ratio of the energy stored in the system to the energy dissipated per cycle. When the Q factor is higher, the rate of energy loss is low, and the resonant frequency peak becomes sharper. The -3 dB points are also referred to as the half power points on the transfer magnitude curve (here is the transmissibility curve).

$$2\zeta = \frac{(\omega_2 - \omega_1)}{\omega_n} \tag{S20}$$

Hourglass sample	-3dB Points (Hz)		Q amplification factor	ζ
Homogeneous auxetic lattice	59.3	76.2	0.25	0.126
Homogeneous solid shell	224	275.6	4.84	0.103
Non-homo. auxetic + solid	100	124.06	4.79	0.104
Non-homo. honeycomb + auxetic	68.7	85	4.7	0.105
Non-homo. honeycomb + solid	94.3	115.3	.505	0.099

Table S3. Experimentally obtained damping values ζ for the homogeneous and non-homogeneous category of hourglass lattice metastructure, using the half-power bandwidth method.

2.6 Tensile testing

The mechanical behavior of PCTPE samples made by 3D printing would certainly depend on various printing parameters such as layer orientation, temperature, infill type, and percentage. Therefore, in this study, a comparative analysis has been performed by fixing all printing constraints same for all samples with 100% infill density and the same orientation on the build platform.

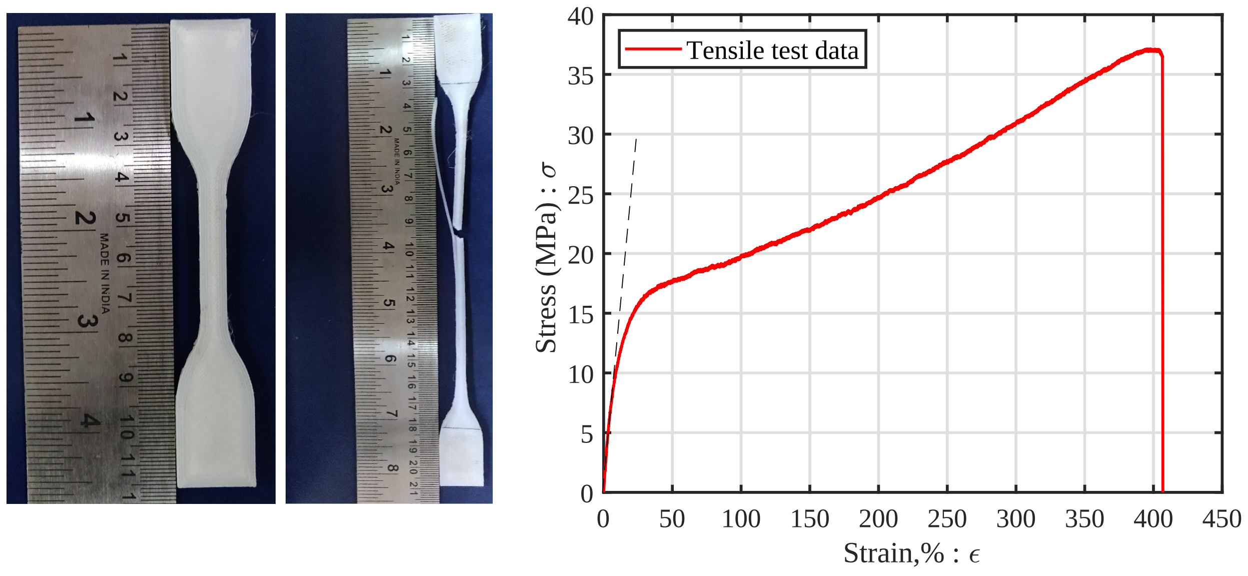

(a) **(b)**

Figure S8. **(a)** Additive layer manufacturing (3D printed) of dogbone shape tensile specimen with the dimensions of ASTM 638-14 Type IV standard (before and after testing images). **(b)** Tensile test data obtained from the universal testing machine (Instron, 100 KN load cell) provides modulus of elasticity (E) shown in a dashed straight line of initial slope and failure occurs on 410 % of elongation.

We have also developed a standard dog bone tensile testing specimen of PCTPE (with dimensions ASTM 638-14 Type IV recommended for polymeric/plastic samples) with 100% infill density and performed testing to evaluate the modulus and its ultimate tensile strength. In comparison of raw PCTPE, the mechanical properties of 3D printed tensile specimen with 100% infill density is shown in below table:

**Specimen type**	**Modulus, E (MPa)**	**Elongation %**
PCTPE filament:<br>Company provided data	73	490
3D printed tensile PCTPE specimen (100% infill):<br>Test data	77	410

Table S4. Tensile testing of standard dogbone specimen with the dimensions of ASTM 638-14 Type IV standard, a comparison table of mechanical properties obtained by tensile testing data of 3D printed 100% infill and the raw PCTPE data provided by the manufacturer.

References

1. Papadopoulou, A., Laucks, J. & Tibbits, S. Auxetic materials in design and architecture. *Nat. Rev. Mater.* **2**, 1–3 (2017).
2. Saxena, K. K., Das, R. & Calius, E. P. Three decades of auxetics research- materials with negative poisson's ratio: a review. *Adv. Eng. Mater.* **18**, 1847–1870 (2016).
3. Evans, K. E. & Alderson, K. Auxetic materials: the positive side of being negative. *Eng. Sci. & Educ. J.* **9**, 148–154 (2000).
4. Witek, A. & Grudzinski, P. Experimental examination of spring-damping properties of plastic materials in forced vibrations conditions. *WIT Transactions on Model. Simul.* **22**, 9 (1999).
5. Crede, C. E. & Ruzicka, J. E. Theory of vibration isolation. *Shock. vibration handbook* **2**, 30–35 (1996).
6. Den Hartog, J. P. *Mechanical Vibrations* (Courier Corporation, 1985).
7. Hussein, M. I., Leamy, M. J. & Ruzzene, M. Dynamics of phononic materials and structures: Historical origins, recent progress, and future outlook. *Appl. Mech. Rev.* **66**, 040802 (2014).
8. Farzbod, F. & Leamy, M. J. Analysis of bloch's method and the propagation technique in periodic structures. *J. Vib. Acoust.* **133**, 031010 (2011).
9. Mark, R. & Hutchinson, P. On the structure of the roman pantheon. *The Art Bull.* **68**, 24–34 (1986).
10. MacDonald, W. L. *The Pantheon: design, meaning, and progeny* (Harvard University Press, 2002).
11. Timoshenko, S. P. & Gere, J. M. *Theory of Elastic Stability* (Courier Corporation, 2009).
12. Almen, J. O. The uniform-section disk spring. *Trans. ASME* **58**, 305–314 (1936).
13. Brecht, W. & Wahl, A. The radially tapered disk spring. *Trans. ASME* **52**, 45–55 (1930).
14. Sadd, M. H. *Elasticity: Theory, Applications, and Numerics* (Academic Press, 2009).
15. Gibson, L. J. & Ashby, M. F. *Cellular Solids: Structure and Properties* (Cambridge University Press, 1999).
